# Synthesis of photoresponsive cholesterol-based azobenzene organogels: dependence on different spacer lengths

**DOI:** 10.3762/bjoc.11.122

**Published:** 2015-06-29

**Authors:** Yuchun Ren, Bin Wang, Xiuqing Zhang

**Affiliations:** 1 Chemical Synthesis and Pollution Control Key Laboratory of Sichuan Province of China, China West Normal University, Nanchong 637009, China

**Keywords:** azobenzene, cholesterol, organogel, photoresponsive

## Abstract

A series of azobenzene–cholesterol organogel compounds (**M_0_–M_12_**) with different spacers were designed and synthesized. The molecular structures were confirmed by ^1^H NMR and ^13^C NMR spectroscopy. The rapid and reversible photoresponsive properties of the compounds were investigated by UV–vis spectroscopy. Their thermal phase behaviors were studied by DSC. The length of the spacer plays a crucial role in the gelation. Compound **M_6_** is the only one that can gelate in ethanol, isopropanol and 1-butanol and the reversible gel–sol transitions are also investigated. To obtain visual insight into the microstructure of the gels, the typical structures of the xerogels were studied by SEM. Morphologies of the aggregates change from flower-like, network and rod with different sizes. By using IR and XRD characterization, it is found that intermolecular H-bonding, the solvents and van der Waals interaction are the main contributions to the specific superstructure.

## Introduction

In the past few decades, low molecular mass organic gelators (LMOGs) have attracted increasing attention not only for basic self-assembly behavior but also for their potential application in areas such as templates [1], light harvesting [2], fluorescent scensing [3], etc. The driving force for the spontaneous formation of gel could be relatively non-covalent interactions such as π–π stacking [4–7], hydrogen bonding [7–9], dipole–dipole [10], and van der Waals interactions [11–12]. A series of cholesterol-based gelators were reported [13–15]. These new classes of organogelator architectures have been systematically studied because of their reversible gel process and unique directional self-association through weak van der Waals interactions [16–17]. Up to now, numerous attempts have been made to develop novel supramolecular architectures, which can bring about new predictable gelation abilities for the construction of novel cholesterol-based gelators.

**Scheme 1:**
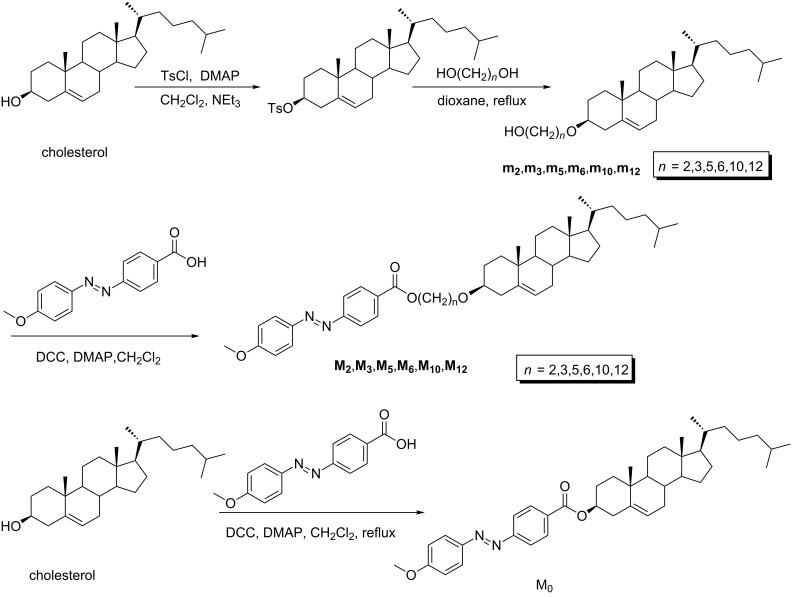
Synthesis route and chemical structure of the compounds.

Azobenzene is one of the smartest molecules among all known photochromic compounds because of its *E*/*Z* isomerization [18]. Based on this, photomechanical soft materials containing azobenzene have been utilized in supramolecular systems [19]. Examples of several cholesterol-linked azobenzenes with gel–sol reversible changes also have been studied [20–23]. R. Zentel found that the photoinduced reversible gel–sol changes between isotropic and anisotropic gels of a class of semicarbazide–azobenzene-based gelators [24]. A new multistimuli photoresponsive organogel containing azobenzene groups was designed and studied by D. Zhu and his co-workers [25]. Q. Zhang synthesized a series of new symmetric dicholesterol-linked gelators [26]. Upon UV–vis irradiation of the gels, a reversible gel–sol transition occurred by breaking and reforming of van der Waals interactions. The cholesterol-substituted diacetylenic polymerized gels exhibit enhanced stability upon thermo- or photo-stimuli [27]. Although similar compounds have been discussed, it is still not very clear about the relationship between the spacer length and the gelation ability of gelators. Herein we synthesized a series of compounds with different spacers between cholesteryl and azobenene units (Scheme 1). In these compounds, **M_6_** is the only one that can gelate in ethanol, isopropanol and 1-butanol. It is found that intermolecular H-bonding, the solvents and van der Waals interaction are the main contributions to the specific superstructure.

## Results and Discussion

**Figure 1:**
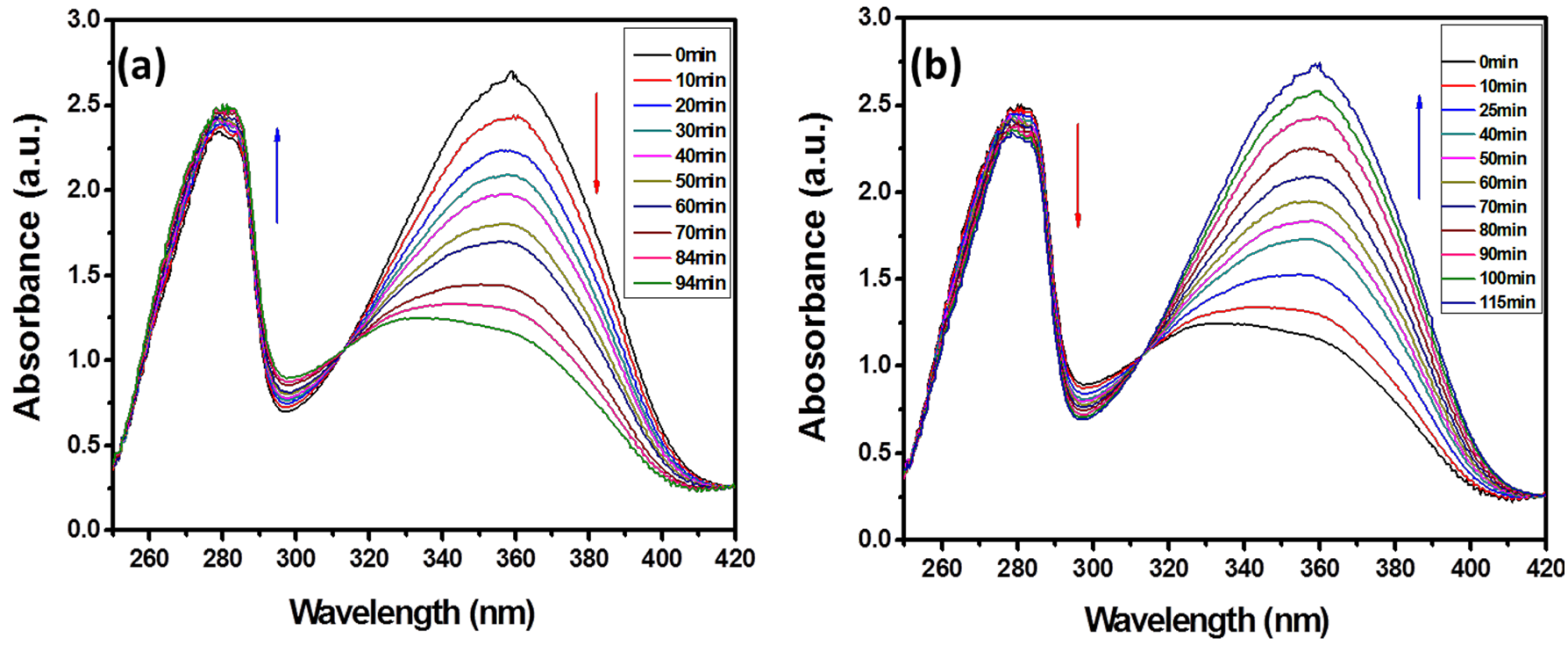
Changes in the absorption over time in the UV–vis spectra of dilute THF solution of M_0_: (a) upon UV-light irradiation (λ = 365 nm) and (b) upon visible-light irradiation (λ = 450 nm) of the solution obtained after irradiation of 365 nm.

To investigate the photoresponsiveness of these compounds, the dilute THF solution of compounds were irradiated by UV and visible light. Figure 1a shows an obvious increase in the peak at 258 nm and a concurrent decrease in the strong peak at 358 nm. The photostationary state was attained after 94 min of irradiation and the spectral changes are attributable to the *trans-*to*-cis* isomerization under UV light irradiation. Further visible light (450 nm) irradiation on the *cis*-solution led to an increase in the absorption intensity at 358 nm and a decrease around 258 nm, and the original spectrum recovered by the reverse *cis-*to*-trans* isomerization (Figure 1b). The absorption band at 358 nm corresponding to the π–π* transition of azobenzene increased, respectively. It should be emphasized that such reversible spectral transformation could be repeated several times. The *trans*-to-*cis* isomerization of the azobenzenze unit had been demonstrated. Compounds **M_2_**, **M_5_**, **M_6_**, and **M_12_** have the same recoverable photoresponsive properties as **M_0_**. Their UV–vis spectra were shown in Supporting Information File 1, Figures S1–S4. **M_3_** and **M_10_** have the photochromic properties.

### Liquid crystal properties of the synthesized compounds

**Figure 2:**
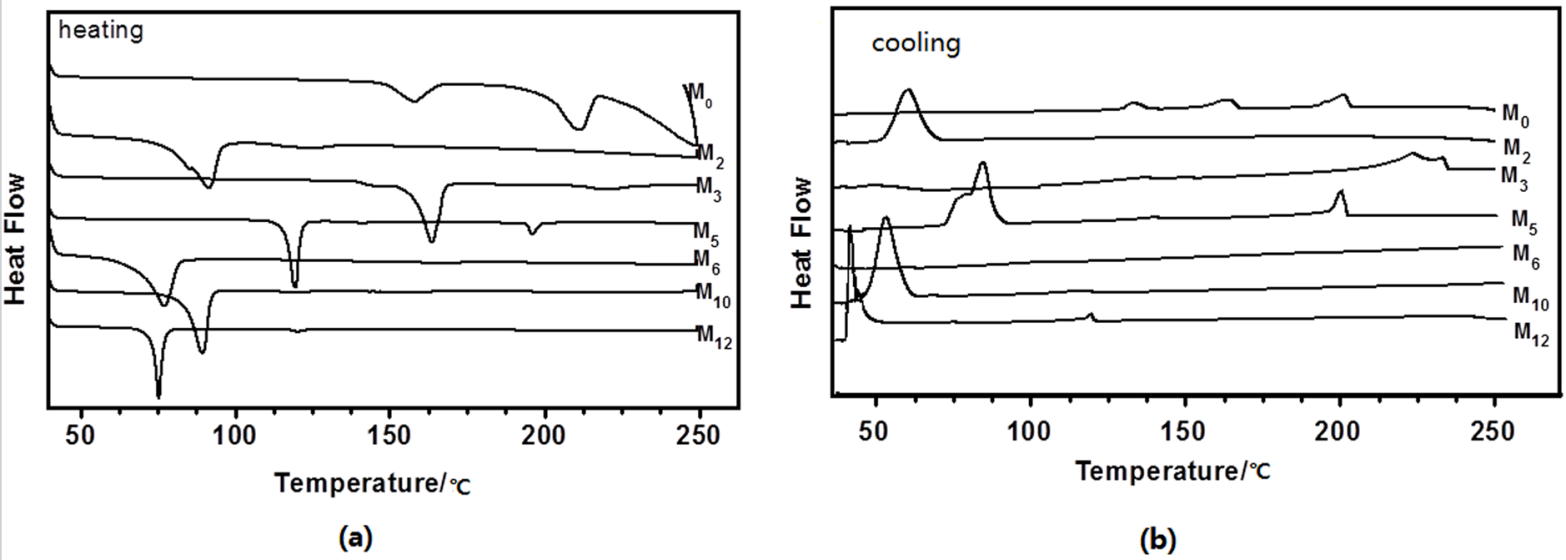
DSC thermogram of the compounds **M_0-n_** obtained on (a) heating and (b) cooling.

The structures of these compounds are shown in Scheme 1. The thermal behaviors of the seven compounds were analyzed by differential scanning calorimetry (DSC). The DSC curves of the cholesterol-based supramolecular complexes are shown in Figure 2 and phase transition temperatures are listed in Table 1. The DSC thermogram of **M_0_** presented three peaks at 158, 211 and 249 °C on heating, corresponding to the crystal to crystal transition, crystal to mesophase transition and mesophase to isotropic transition. On the cooling run, DSC curves presented isotropic phase to mesophase, mesophase to crystal and crystal to crystal at 190, 155 and 126 °C. Each curve of compounds **M_2_**, **M_6_** and **M_10_** displayed a single endothermic melting peak. The heating DSC curves of **M_3_**, **M_5_** and **M_12_** contained two endothermic peaks. The peaks at lower temperature were due to the crystal to mesophase transitions, and the second peaks displayed the mesophase to isotropic transitions. The DSC thermogram of **M_3_**, **M_5_** and **M_12_** were observed two exothermic peaks on cooling process, which indicated the transitions from isotropic phase to mesophase at higher temperature and mesophase to crystal transitions at lower temperature

**Table 1:** Phase transition temperatures of the compounds.

sample	heating (°C)	cooling (°C)

**M_0_**	K 158 K_2_ 211 N 249 I	I 190 N 155 K_1_ 126 K_2_
**M_2_**	K 92 I	I 59 K
**M_3_**	K 164 N 220 I	I 224 N 213 K
**M_5_**	K 120 N 197 I	I 193 N 83 K
**M_6_**	K 78 I	—
**M_10_** **M_12_**	K 90 I K 75 N 120 I	I 54 K I 120 N 42 K

K: crystalline state, N: nematic phase, I: isotropic liquid.

### Gelation behaviors

**Figure 3:**
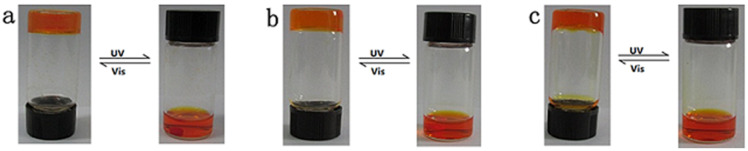
Photographic images of the **M_6_** sol-gel transition behavior after UV or visible light irradiation in different solution (a) ethanol, (b) isopropanol and (c) 1-butanol.

The gelation behaviors of the seven compounds were tested in 22 solvents at room temperature and the results were listed in Table 2. Among the compounds examined, **M_6_** is the only one that forms a gel in ethanol, 1-butanol and isopropanol, while the other compounds cannot gelate in any solvent. Generally speaking, the gels were formed by a heating and cooling cycle process. Examination of the data shown in the table reveals that the change of spacer length in the molecular skeleton has a significant effect on the gelation abilities of the compounds. To investigate the photoresponsiveness of the three gels, we performed the photoirradiation of gels using a UV lamp. The formed gels at room temperature were in an opaque state. Furthermore, the gels with both solvents are very stable for months of storage in a closed container, and no significant changes can be found. However, upon UV irradiation, the gel to sol phase transition became transparent as a result of *trans-*to-*cis* isomerization. Moreover, the solution has a tendency to reverse the sol to gel phase transition under visible irradiation. The corresponding visual images are shown in Figure 3.

**Table 2:** Gelation performances of the seven cholesteryl derivatives at room temperature.

Solvent	**M_0_**	**M_2_**	**M_3_**	**M_5_**	**M_6_**	**M_10_**	**M_12_**

Methanol	I	I	I	I	I	I	I
Ethanol	I	I	I	I	G	I	I
1-Propanol	I	I	I	I	I	I	I
1-Butanol	I	I	I	P	G	I	I
1-Pentanol	I	I	I	I	I	I	I
1-Octanol	I	P	I	I	I	I	I
1,4-Butanediol	I	I	I	I	I	I	I
Isopropanol	I	I	I	I	G	I	I
Acetone	I	P	I	P	S	I	P
DMSO	I	I	I	I	I	I	I
DMF	I	P	I	I	S	I	I
EtOAc	I	S	I	P	S	P	P
Kerosene	I	P	I	I	I	P	S
Benzene	S	S	S	S	S	S	S
Toluene	S	S	S	S	S	S	S
Xylene	S	S	S	S	S	S	S
THF	S	S	S	S	S	S	S
*n*-Hexane	I	P	I	P	P	P	P
Dichloromethane	S	S	S	S	S	S	S
Chloroform	S	S	S	S	S	S	S
Tetrachloromethane	S	S	S	S	S	S	S
Distilled water	I	I	I	I	I	I	I

G: turbid gel; S: solution; P: precipitate; I: insoluble.

### Scanning electron microscopy studies

**Figure 4:**
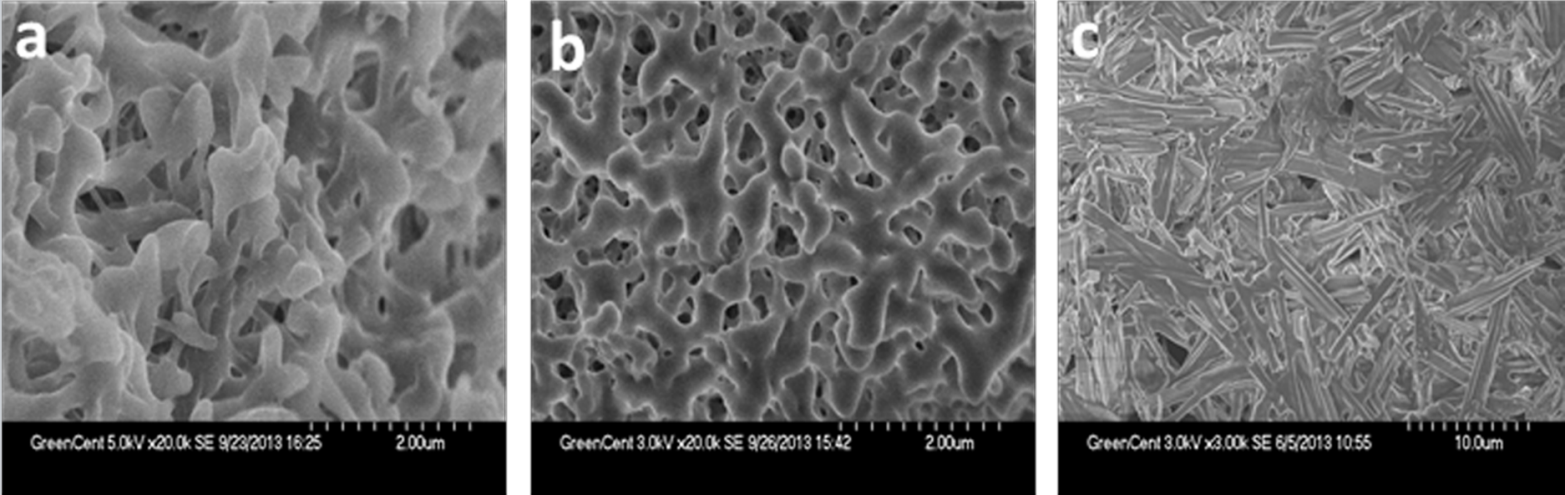
SEM images of xerogels formed with **M**_6_ from solution: (a) ethanol, (b) isopropanol and (c) 1-butanol.

To obtain a visual insight into the morphology of the aggregation mode, the typical structures of the xerogels prepared by the freeze-drying of the three gels in different solvents were studied by SEM (Figure 4). With the reference to the images, it can be clearly seen that the structures of the three gelators are significantly different from each other. The morphologies of the aggregates change from flower-like to network and rod with different sizes. Obviously, the gel from ethanol mainly shows a similar flower-like morphology. When we focused on the isopropanol xerogel, we could see that there are many regularly three-dimensional network structures. In 1-butanol, the organogelator molecules in the gel phase were self-assembled into tightly staked rods. Different solvents caused a sharp difference between these morphologies of the xerogels.

### The FTIR spectroscopy

**Figure 5:**
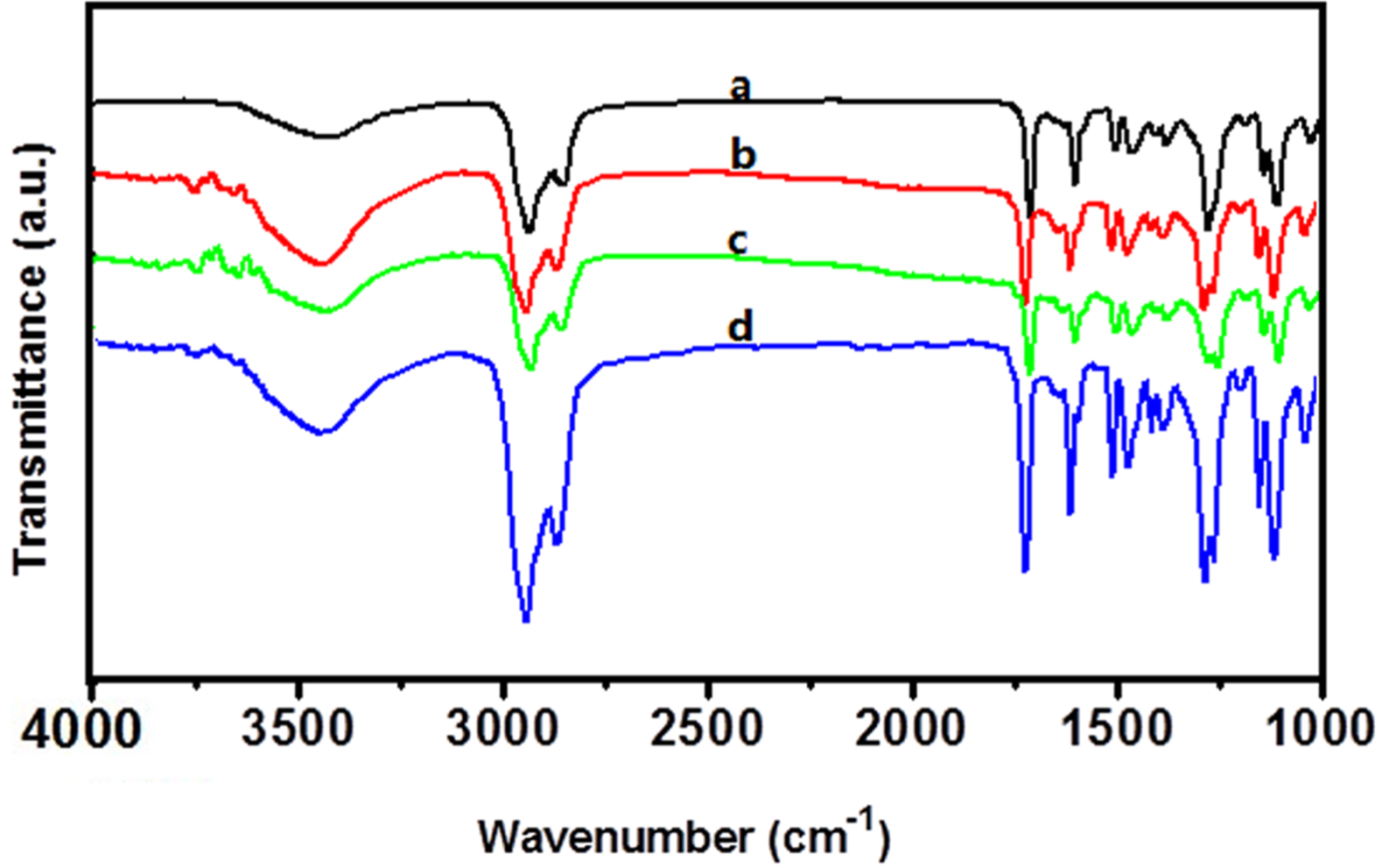
FTIR spectra of gels**_:_** (a) powder, (b–d) ethanol, isopropanol and 1-butanol xerogels, respectively.

It should be emphasized that the organogels achieved through strength and directionality of hydrogen bonding. Further information about the intermolecular interactions of these gels was obtained from the FTIR spectroscopy. Figure 5 shows the IR spectra of **M_6_** powder and its gels in ethanol, isopropanol and 1-butanol. Gels show two peaks which located at 3740 and 3672 cm^−1^, characteristic of the hydrogen bonding O–H stretching vibration. As for the powder, the absorption bands cannot be found in the same vibrational area. The peak observed at 3437cm^−1^ can be assigned to the intermolecular association hydrogen stretching vibration. However, the band of ethanol, isopropanol and 1-butanol shifted to 3438, 3439 and 3440 cm^−1^, respectively. The comparison of the IR spectra of powder and gels indicated the hydrogen-bond interaction in the gels. The bands of the asymmetric and symmetric CH_2_ stretching vibration of **M_6_** powder emerged at 2935 and 2852 cm^−1^, while, in the ethanol gel, they shifted to 2934 and 2858 cm^−1^, in isopropanol gel, they shifted to 2934 and 2862 cm^−1^, in 1-butanol , they shifted to 2933 and 2859 cm^−1^. The above mentioned FTIR spectral changes demonstrated sufficiently that van der Waals interaction between the alkyl chains plays an important role in the self-assembly process of organogels.

### The XRD studies

**Figure 6:**
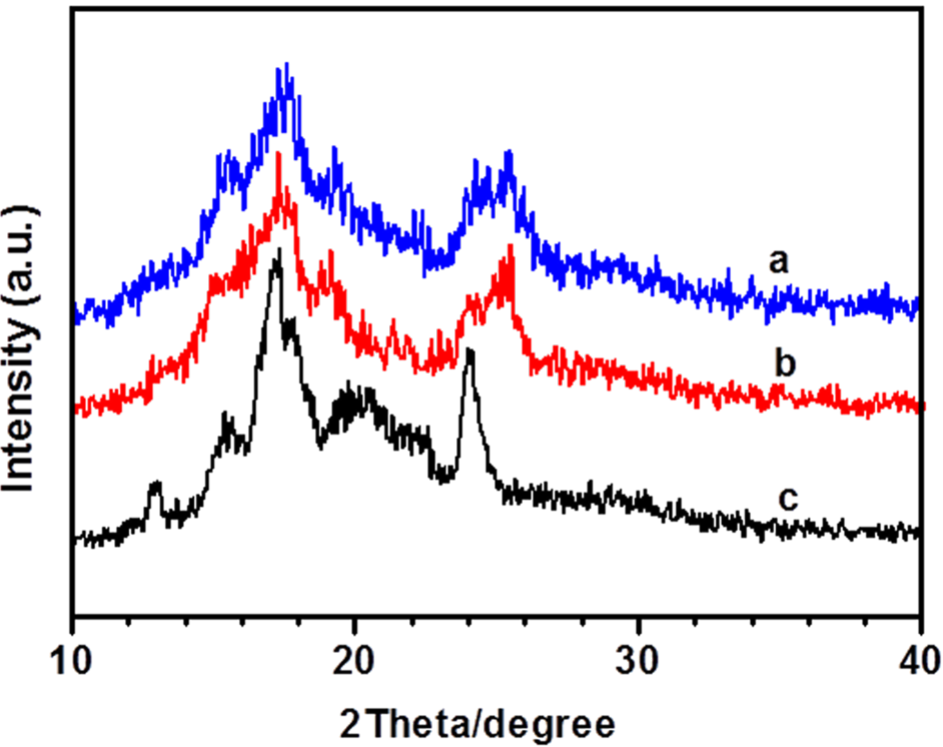
XRD patterns of xerogels. (a) ethanol, (b) isopropanol and (c) 1-butanol.

To reveal the detailed changes in gel molecular of **M_6_**, XRD analyses were conducted and the results were displayed in Figure 6. The pattern of the xerogel obtained from the 1-butanol gel exhibited five peaks at 12.84, 15.38, 17.22, and 20.44°, corresponding to *d*-spacing of 6.90, 5.80, 5.14, 4.30, and 3.70 nm, respectively. The *d*-spacing almost in ratio of 7:6:5:4, may due to the molecular arrangement with order or existence layers of the stacking between molecular. The last peak may relate to the periodic intermolecular distances at different direction and intermolecular separations. Two diffraction peaks at 17.32° and 25.40° were detected for the xerogels of another two solvents, corresponding to *d* values of 5.10 and 3.50 nm, respectively. The different self-assembly modes of gelator in various solvents cause the values changing. The XRD results described above also demonstrated that the molecular interaction and the solvents had a significant impact on the gel formation modes.

## Experimental

### Materials

The starting materials, thylene glycol, 1,3-propanediol, 1,5-pentadiol, 1,6-hexanediol, 1,10-decanediol, 1,12-dodecanediol, cholesterol, 4-toluene sulfonyl chloride, phenol, 4-aminobenzoic acid, iodomethane, sodium nitrite, hydrochloric acid, sodium hydroxide, *N*,*N*'-dicyclohexylcarbodiimide (DCC), 4-dimethylaminopyridine (DMAP), all were purchased from Sigma-Aldrich Chemicals (Shanghai, China). Other used reagents were commercially available and were distilled before used. The synthesis of the 4'-carboxy-4-methoxyazobenzene ultilized in this study was described elsewhere [28].

### General measurements

NMR spectra were recorded with a Bruker-400 NMR spectrometer. Powder X-ray diffraction (XRD) measurements were carried out on a Rigaku D/max 2500PC (Japan)-type single-crystal diffractometer using Cu Kα radiation to determine the crystal structure. Infrared spectroscopy (IR) was measured on a Nicolet 6700 spectrometer from 4000 to 500 cm^−1^ at room temperature. The samples were prepared as KBr pellets. The morphology of the gel samples were examined by scanning electron microscopy (SEM, S5200, FEI Company) under an acceleration voltage of 30 kV. UV–vis absorption spectra were recorded using a spectrophotometer (UV-3600, Shimadzu, Japan). Differential scanning calorimetry (DSC) was carried out on a thermal analysis (TA) DSC-Q30 in nitrogen at a heating rate of 10 K/min.

### Synthetic procedures

**Synthesis of m_2_–m_12_.** 3β-[(2-hydroxyethyl)oxy]cholest-5-ene (**m_2_**) was synthesized similar to a previous report [29]. Cholesterol 15.00 g (38 mmol) in dry dichloromethane (225 mL) and dry triethylamine (16.50 mL), *p*-tosyl chloride 10.35 g (54 mmol) was added. A catalytic amount of DMAP was also added. The reaction mixture was then allowed to stir at 48 °C for 12 h. The reaction mixture was washed sequentially with diluted HCl aqueous solution, saturated brine solution, and water. The organic phase was dried over anhydrous Na_2_SO_4_. The solvents were removed by vacuum evaporation and recrystallized using chloroform and methanol. The residue (7.0 g, 13 mmol) was dissolved in anhydrous dioxane (60 mL) and dry ethylene glycol (20 g, 0.32 mol) was added. The mixture was refluxed at 110 °C for 4 h. The solution was cooled and solvent was removed under vacuum. The white residue was dissolved in chloroform. The organic layer washed with NaHCO_3_, water, and brine; and dried over anhydrous Na_2_SO_4_. Finally, the product was purified by column chromatography over silica gel eluting with petroleum ether/ethyl acetate to yield 90% of **m_2._** The procedure used for the preparation of **m_3_**, **m_5_**, **m_6_**, **m_10_**, **and m_12_** are similar to that for **m_2_**.

### 4-Methoxy-4’-((cholesteryloxy)carbonyl)azobenzene (**M_0_**)

A mixture of 4'-carboxy-4-methoxyazobenzene (0.25 g, 10 mmol), cholesterol (0.38 g, 1 mmol), and DCC (0.21 g, 1 mmol), DMAP (0.06 g, 0.5 mmol) in 25 mL CH_2_Cl_2_ was stirred at room temperature for 24 h. The solvent was evaporated under vacuum. The product was purified by column chromatography over silica gel eluting with pure CH_2_Cl_2_, yield 70%. Related compounds (**M_2_**, **M_3_**, **M_5_**, **M_6_**, **M_10_**, **M_12_**) were synthesized by a similar method.

### Gelation test

The potential gelator (25 mg) in solvent (1 mL) was placed in a sealed test tube and the solution was heated in a water bath until the solid was dissolved completely. The solution was cooled to room temperature. If the gelator formed a gel at this stage, it was classified as “G”. When the solution remained all the time, they were referred to as solutions (S). The hot clear solution systems, which precipitated when they are cooled down to room temperature, are donated as P (precipitation), respectively. Insoluble systems in which the potential gelator could not be dissolved even at the boiling point of the solvent were designated as insoluble (I).

## Conclusion

In summary, we have synthesized seven cholesterol-based compounds with azobenzene groups. The compounds with different flexible alkyl spacers containing two, three, five, six, ten, or twelve carbon atoms are denoted as **M_2_–M_12_**, respectively. Upon UV–vis irradiation, these compounds can undergo a typical reversible *trans*–*cis* and *cis*–*trans* change due to the photo-isomerization of the azobenzene units. They possess recoverable photoresponsive properties. Their thermal phase behaviors have been investigated by DSC, indicating good liquid crystal properties. The dramatic change in the gelation behavior was produced by the different spacer lengths between cholesteryl and azobenzene. Compound **M_6_** is the only good gelator that can gelate in ethanol, isopropanol and 1-butanol. Based on the results of SEM, IR and XRD, it demonstrated that the solvents, intermolecular H-bonding, and van der Waals interaction affected the aggregation mode and morphology of gels. These results can be applied in fields of design and development of novel organogelators and soft matter.

## Supporting Information

File 1 Spectroscopical and analytical data.
