# Supplementary material for: Synthesis of photoresponsive cholesterol-based azobenzene organogels: dependence on different spacer lengths
Source: Beilstein J Org Chem. 2015 Jun 29;11:1089–95. doi: 10.3762/bjoc.11.122 (PMC4505088; doi:10.3762/bjoc.11.122)

Supporting Information

for

Synthesis of photoresponsive cholesterol-based azobenzene organogels: dependence on different spacer lengths

Yuchun Ren, Bin Wang* and Xiuqing Zhang

Address: Chemical Synthesis and Pollution Control Key Laboratory of Sichuan Province of China, China West Normal University, Nanchong 637009, China

Email: Bin Wang - cwnuwangb@126.com

*Corresponding author

Spectroscopical and analytical data

The UV–vis spectra of compounds M_2, M_5, M_6, and M_{12}

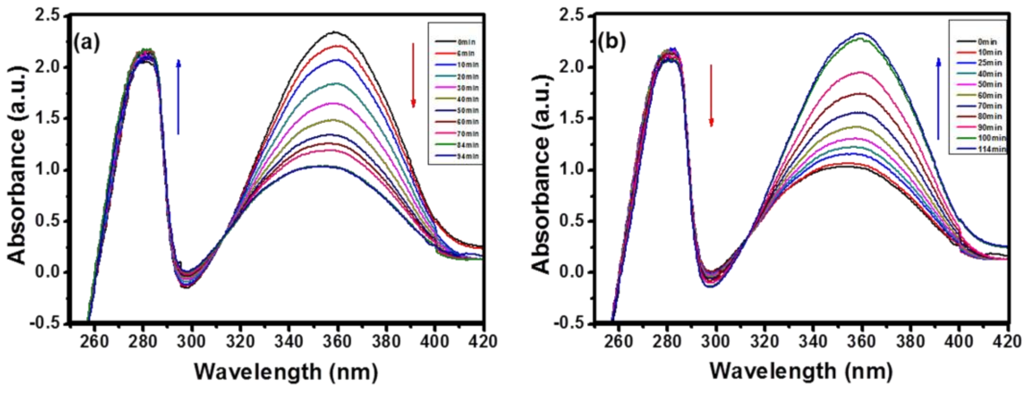

Figure S1: Changes in the absorption over time in the UV–vis spectra of dilute THF solution of $\mathbf{M_2}$: (a) upon UV-light irradiation(λ = 365 nm) and (b)upon visible-light irradiation λ = 450 nm) of the solution obtained after irradiation of 365 nm.

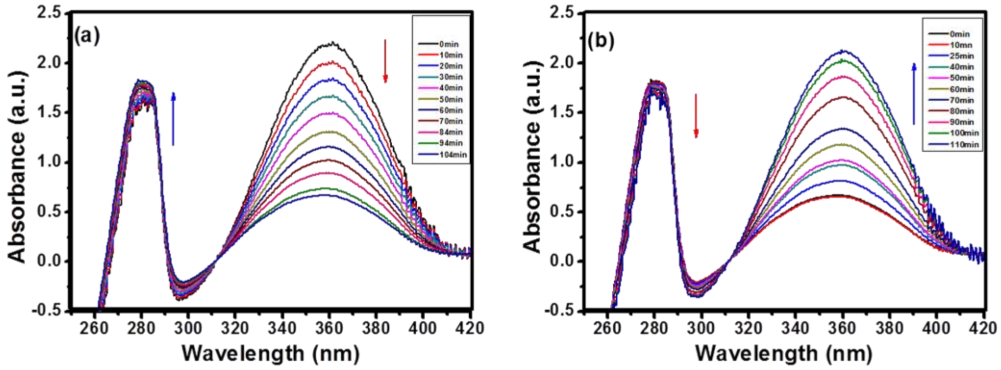

Figure S2: Changes in the absorption over time in the UV–vis spectra of dilute THF solution of **M_5**: (a) upon UV-light irradiation(λ = 365 nm) and (b)upon visible-light irradiation λ = 450 nm) of the solution obtained after irradiation of 365 nm.

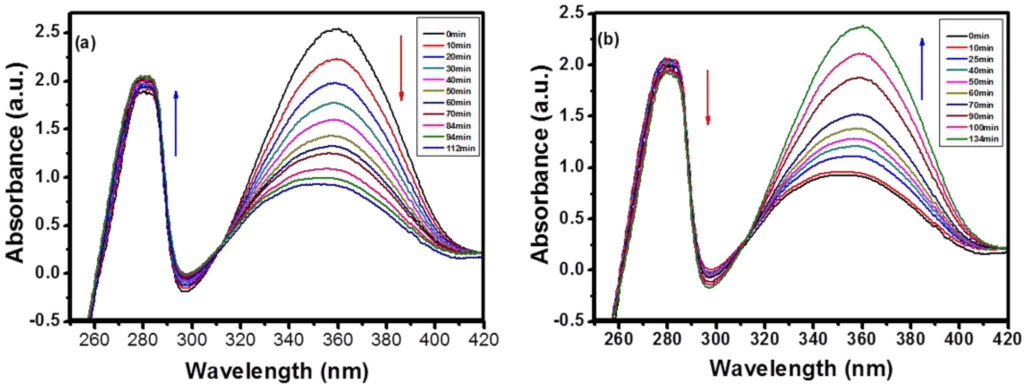

Figure S3: Changes in the absorption over time in the UV–vis spectra of dilute THF solution of **M_6**: (a) upon UV-light irradiation(λ= 365 nm) and (b)upon visible-light irradiation λ = 450 nm) of the solution obtained after irradiation of 365 nm.

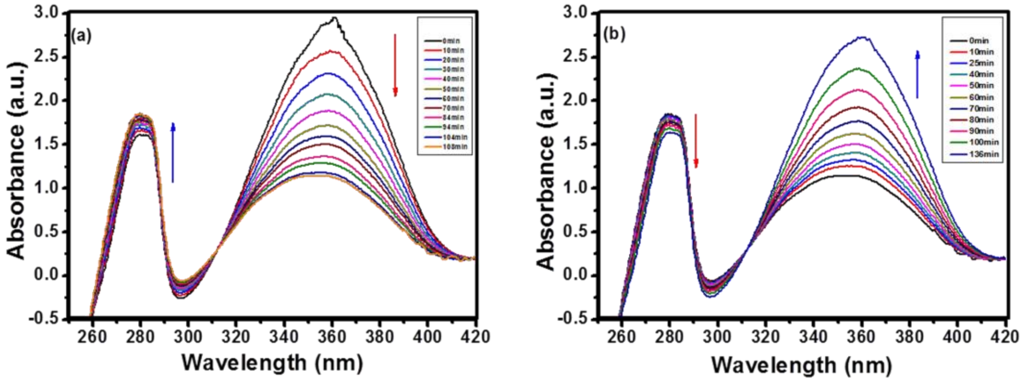

Figure S4: Changes in the absorption over time in the UV-vis spectra of dilute THF solution of **M_{12}**: (a) upon UV-light irradiation(λ= 365 nm) and (b)upon visible-light irradiation λ=450 nm) of the solution obtained after irradiation of 365 nm.

Basic characterization of compounds

^{1}H NMR of m_2: ^{1}H NMR (400 MHz, $CDCl_3$) δ 5.35 (d, *J* = 8.0 Hz, 1H), 3.72 (dd, *J* = 8.0, 4.0 Hz, 3H), 3.59 (t,J=4.0 Hz, 2H), 3.19 (m, 1H), 2.37 (dd, *J* = 12.0, 4.0Hz, 1H), 2.21 (t, *J* = 11.2 Hz, 1H), 2.03 – 1.06 (m, 26H), 1.00 (d, J=8 Hz, 3H), 0.91 (d, *J* = 8.0 Hz, 3H), 0.86 (dd, *J* = 6.6, 1.3 Hz, 6H), 0.67 (s, 3H).

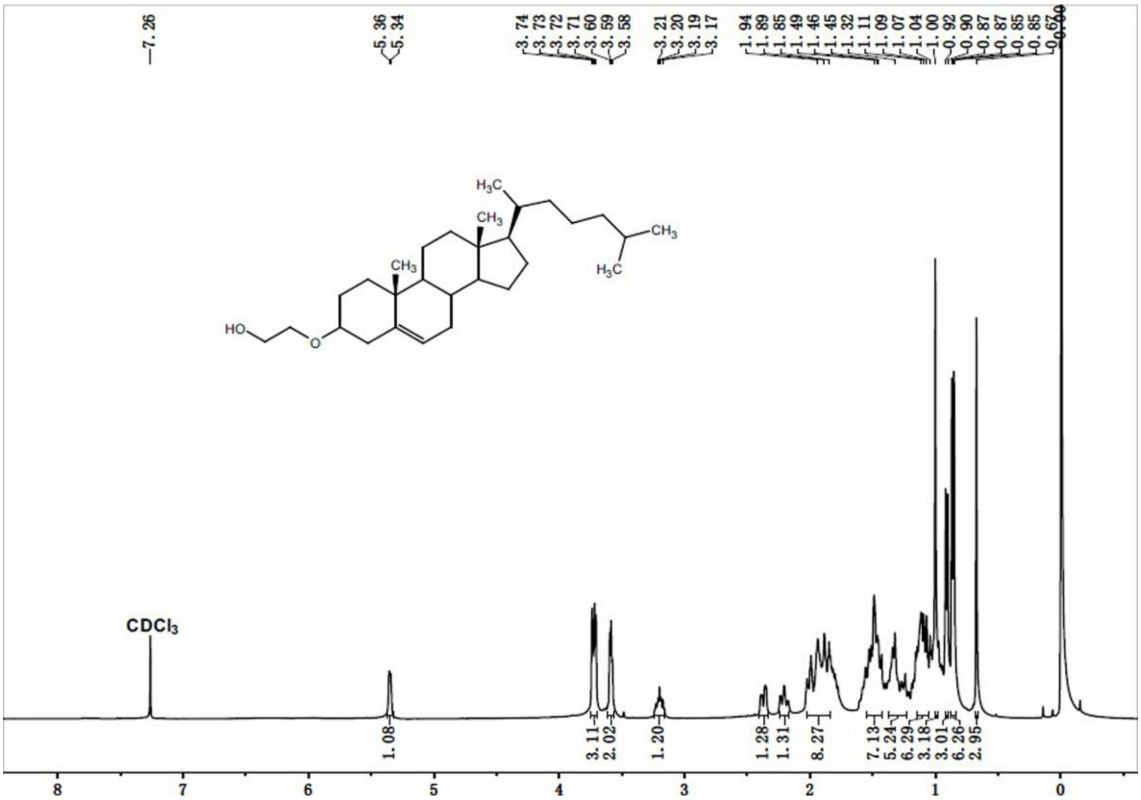

^{1}H NMR of m$_3$: ^{1}H NMR (400 MHz, $CDCl_3$) δ 5.34 (d, *J* = 4.0 Hz, 1H), 3.78 (t, *J* = 4.0 Hz, 2H), 3.68 (t, *J* = 6.0 Hz, 2H), 3.20 – 3.13 (m, 1H), 2.37 (dd, *J* = 16.0, 4.0 Hz, 1H), 2.21 – 2.15 (m, 1H), 1.90 – 1.01 (m, 29H), 0.99 (s, 3H), 0.91 (d, *J* =8.0 Hz, 3H), 0.88 – 0.84 (m, 6H), 0.67 (s, 3H).

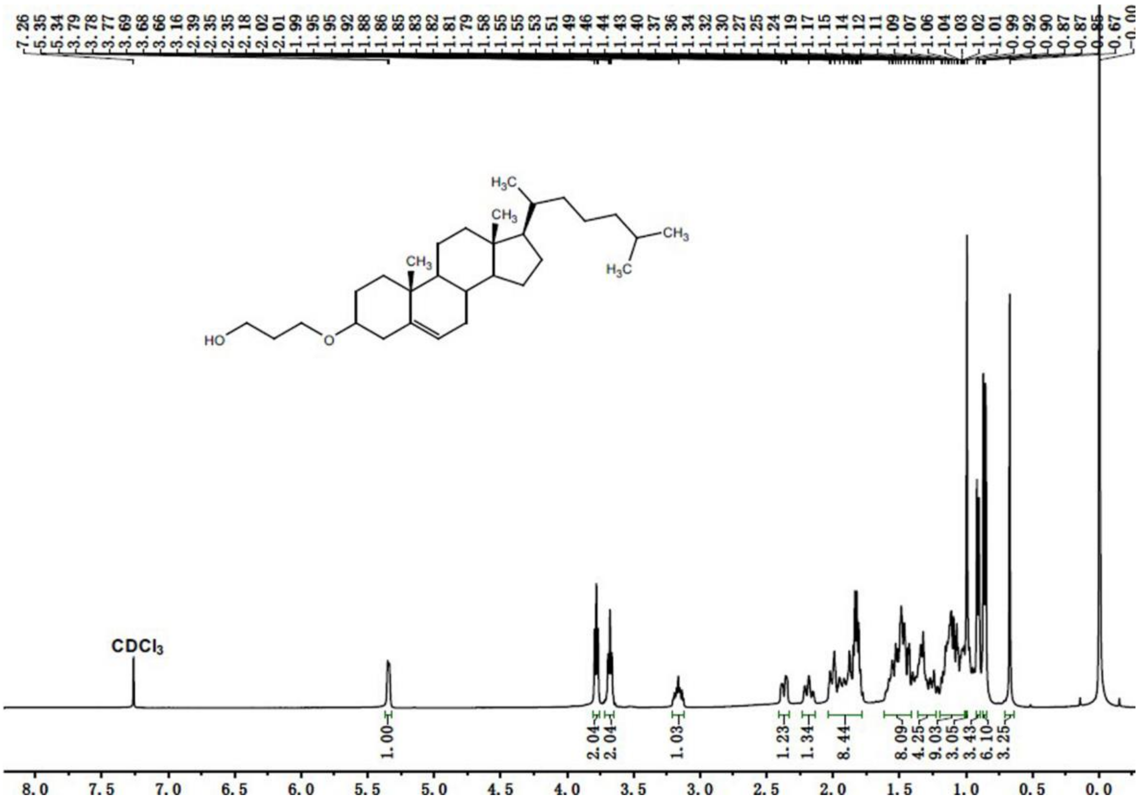

¹H NMR of m_5: ¹H NMR (400 MHz, $CDCl_3$) δ 5.34 (s, 1H), 3.64 (t, *J* = 6.4 Hz, 2H), 3.46 (t, *J* = 6.1 Hz, 2H), 3.12 (m, *J* = 15.4, 5.5 Hz, 1H), 2.35 (dd, *J* = 13.0, 2.6 Hz, 1H), 2.18 (t, *J* = 11.4 Hz, 1H), 2.06– 1.05 (m, 33H), 0.99 (s, 3H), 0.91 (d, *J* = 6.5 Hz, 3H), 0.87 – 0.84 (m, 6H), 0.67 (s, 3H).

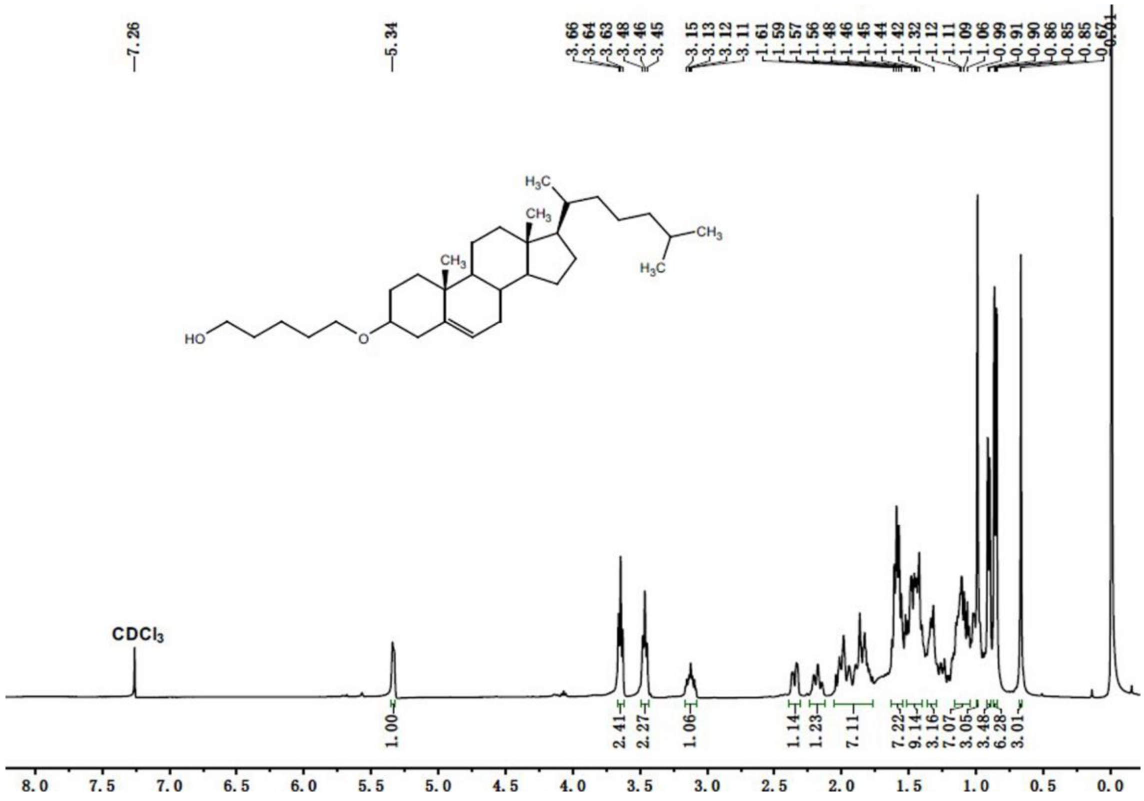

^{1}H NMR of m_6: ^{1}H NMR (400 MHz, $CDCl_3$) δ 5.34 (m, 1H), 3.64 (t, *J* = 6.0 Hz, 2H), 3.45 (t, *J* = 6.0 Hz, 2H), 3.15-3.08 (m, 1H), 2.35 (dd, *J* = 13.1, 2.6 Hz, 1H), 2.18 (t, *J* = 11.3 Hz, 1H), 2.05 –1.05 (m, 35H), 0.99 (s, 3H), 0.91 (d, *J* = 6.5 Hz, 3H), 0.86 (dd, *J* = 6.6, 1.3 Hz, 6H), 0.67 (s, 3H).

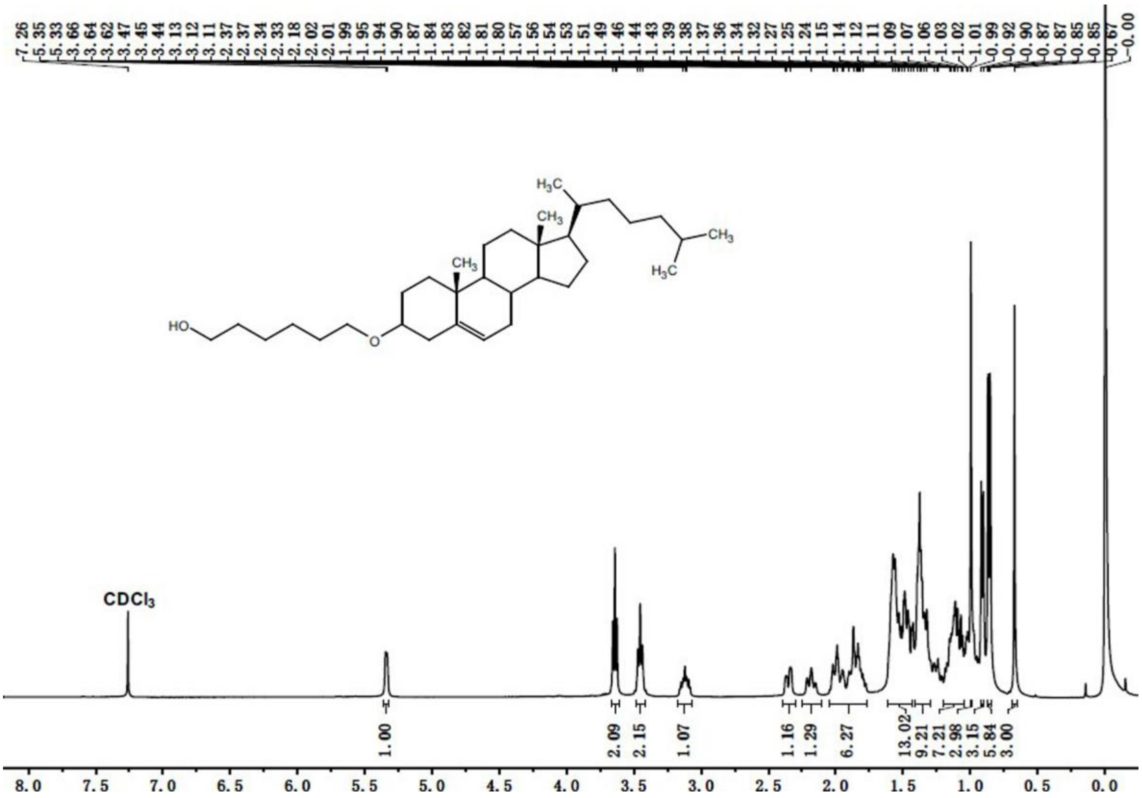

^{1}H NMR of m_{10}: ^{1}H NMR (400 MHz, $CDCl_3$) δ 5.33 (s, 1H), 4.04 (t, *J* = 6.7 Hz, 1H), 3.63 (d, *J* = 6.5 Hz, 2H), 3.43 (t, *J* = 6.6 Hz, 2H), 3.18 – 3.05 (m, 1H), 2.35 (d, *J* = 13.0 Hz, 1H), 2.17 (t, *J* = 12.0 Hz, 1H), 2.08 –1.01 (m, 42H), 0.99 (s, 3H), 0.90 (d, *J* = 6.3 Hz, 3H), 0.85 (d, *J* = 6.2 Hz, 6H), 0.66 (s, 3H).

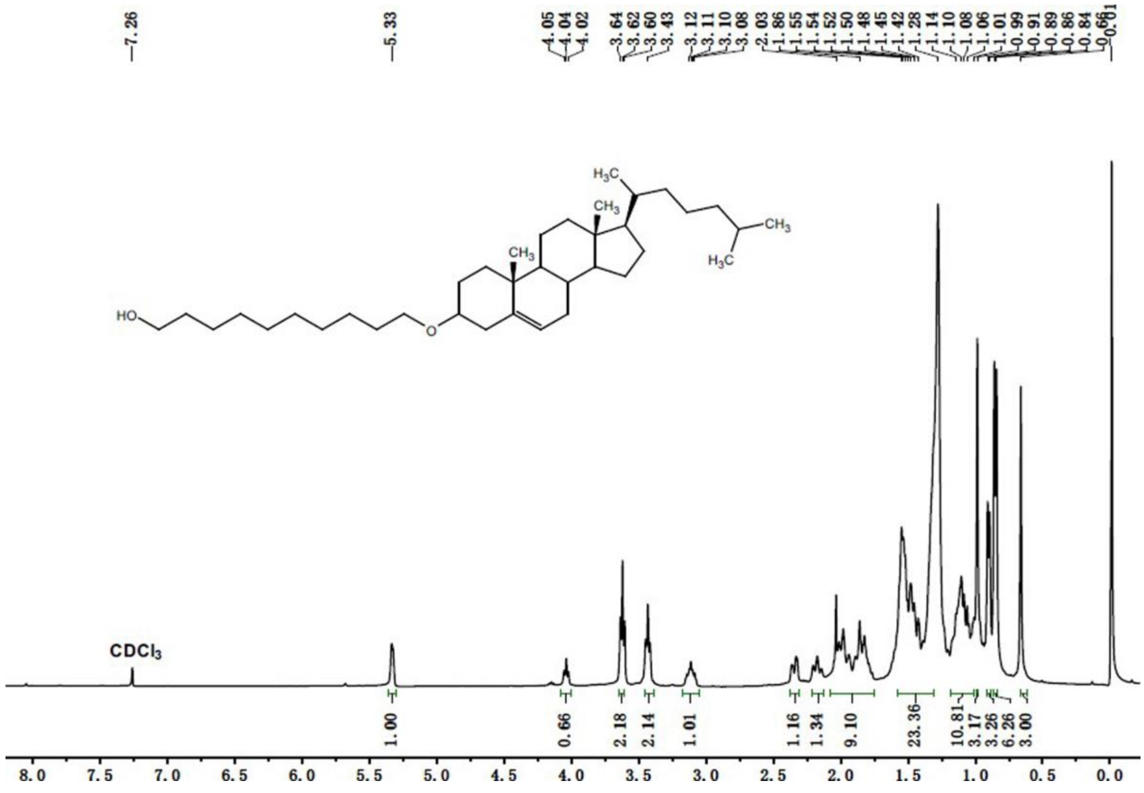

^{1}H NMR of m_{12}: ^{1}H NMR (400 MHz, $CDCl_3$) δ 5.35 (s, 1H), 3.64 (t, *J* = 6.6 Hz, 3H), 3.44 (t, *J* = 6.8 Hz, 2H), 3.11 (m, *J* = 15.3, 5.5 Hz, 1H), 2.35 (dd, *J* = 12.9, 2.9 Hz, 1H), 2.18 (t, *J* = 12.1 Hz, 1H), 1.90– 1.04 (m, 46H), 0.99 (s, 3H), 0.91 (d, *J* = 6.4 Hz, 3H), 0.86 (d, *J* = 6.6 Hz, 6H), 0.67 (s, 3H).

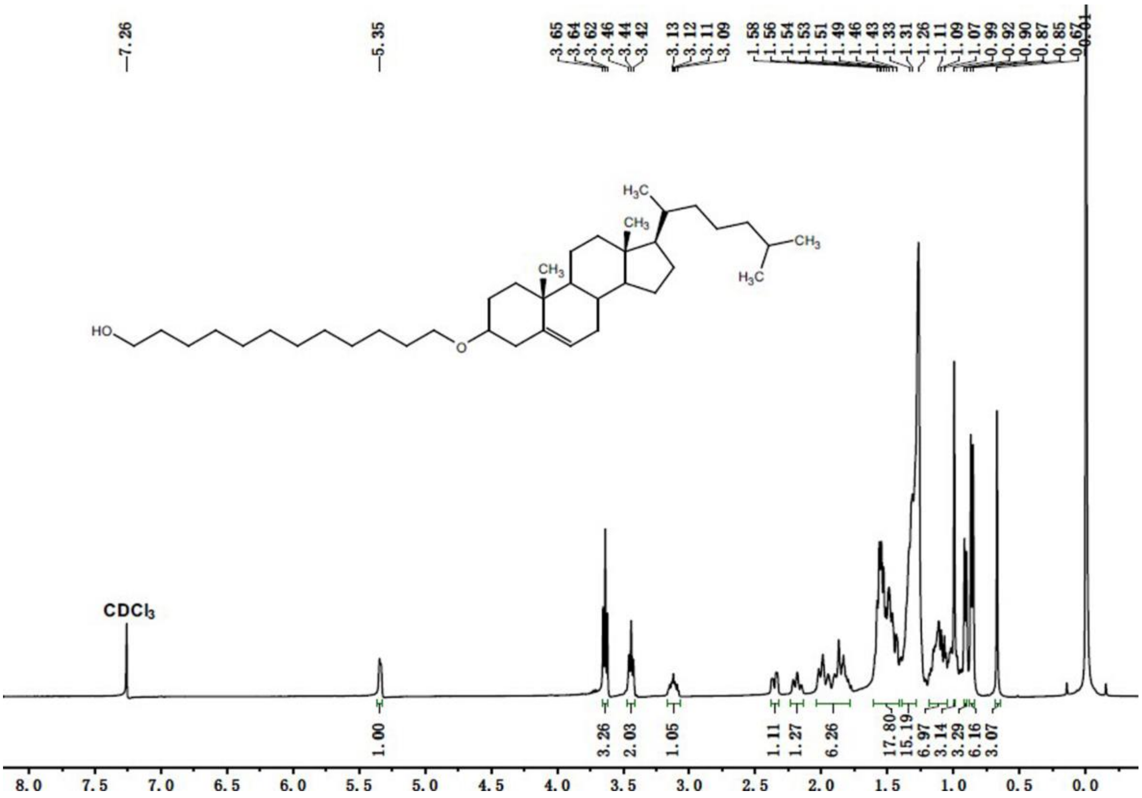

^{1}H NMR of M_0: ^{1}H NMR (400 MHz, $CDCl_3$) δ 8.20 – 8.12 (d, 2H), 7.98 – 7.93 (d, 2H), 7.90 (d, *J* = 8.7, 2.0 Hz, 2H), 7.06 – 7.00 (m, 2H), 5.44 (d, *J* = 3.6 Hz, 1H), 4.89 (m, *J* = 11.8, 10.3, 6.5 Hz, 1H), 3.91 (s, 3H), 2.49–1.24 (m, 28H), 1.08 (s, 3H), 0.93 (d, *J* = 6.5 Hz, 3H), 0.87 (dd, *J* = 6.6, 1.8 Hz, 6H), 0.69 (s, 3H).

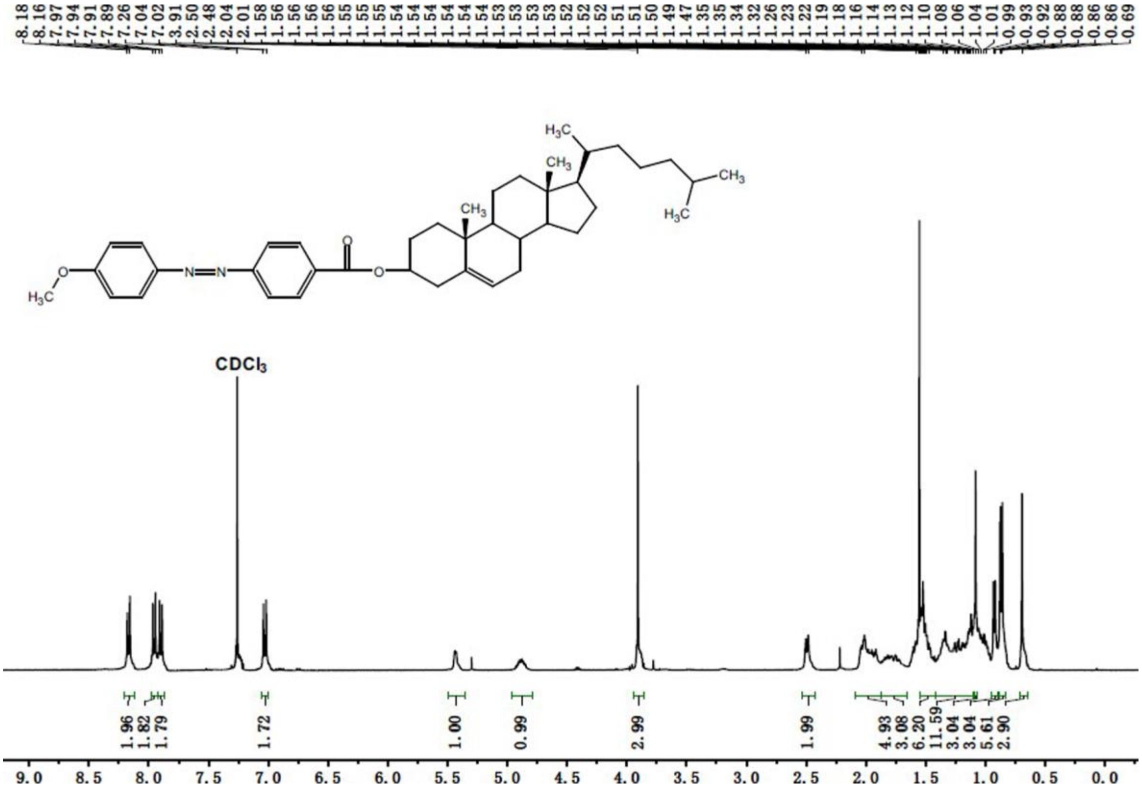

^{13}C NMR of M_0 ^{13}C NMR (101 MHz, $CDCl_3$) δ 165.54, 162.64, 156.72, 155.28, 147.07, 139.64, 131.94, 130.53, 125.17, 122.85, 122.28, 114.32, 74.89, 56.72, 56.17, 55.62, 50.09, 49.15, 42.35, 39.77, 39.53, 38.24, 37.06, 36.68, 36.20, 35.80, 33.97, 31.91, 29.69, 28.23, 28.01, 27.91, 25.63, 24.94, 24.30, 23.84, 22.80, 22.55, 21.07, 19.38, 18.73, 11.87.

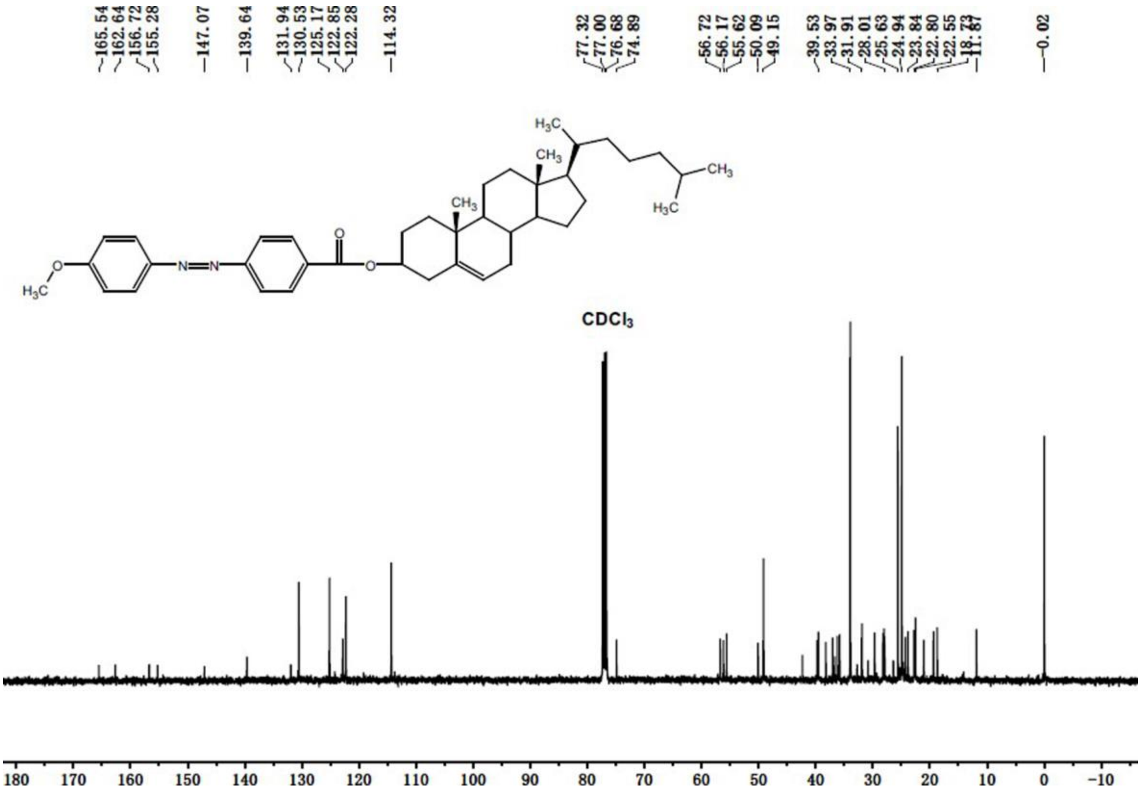

^{1}H NMR of M$_2$: ^{1}H NMR (400 MHz, $CDCl_3$) δ 8.19 (d, *J* = 8.3 Hz, 2H), 7.96 (d, *J* = 9.0 Hz, 2H), 7.91 (d, *J* = 8.3 Hz, 2H), 7.03 (d, *J* = 8.9 Hz, 2H), 5.35 (d, *J* = 5.3 Hz, 1H), 4.51 – 4.43 (m, 2H), 3.93 – 3.81 (m, 5H), 3.31 – 3.18 (m, 1H), 2.40 – 1.04 (m, 28H), 1.01 (s, 3H), 0.91 (d, *J* = 6.3 Hz, 3H), 0.86 (dd, *J* = 6.6, 1.6 Hz, 6H), 0.68 (s, 3H).

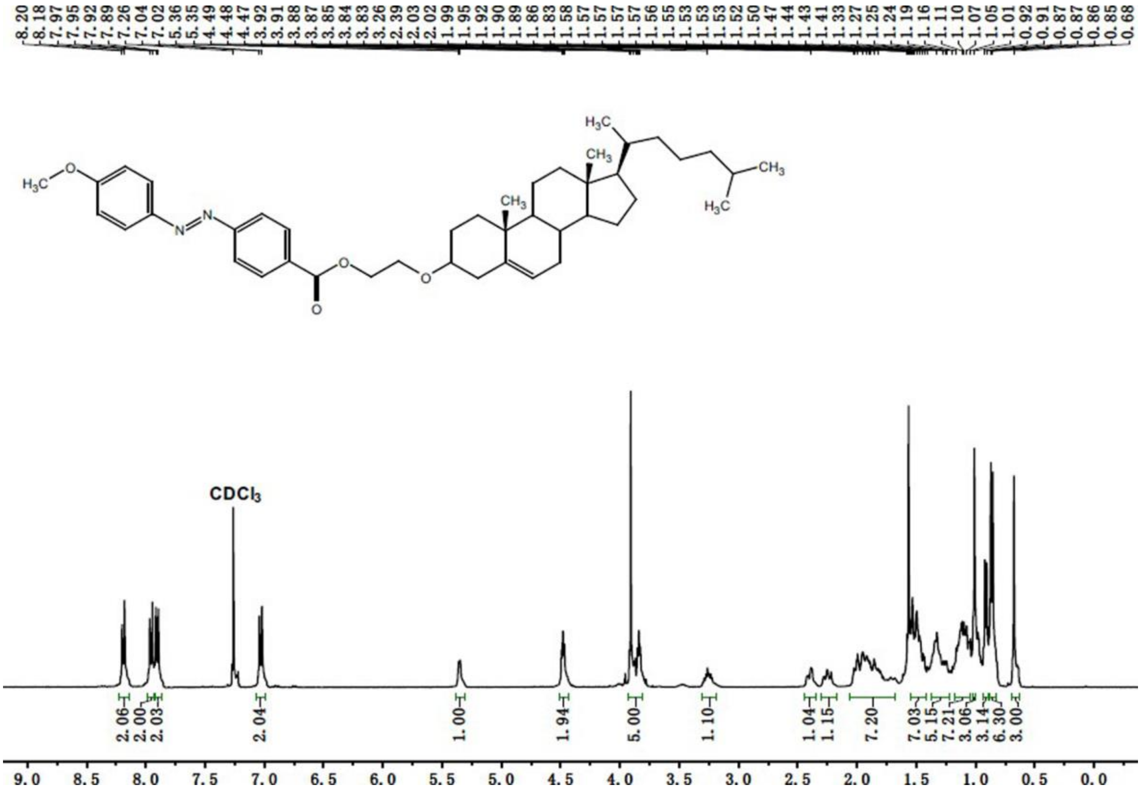

^{13}C NMR of M$_2$: ^{13}C NMR (101 MHz, $CDCl_3$) δ 166.13, 162.68, 155.42, 147.07, 140.79, 131.26, 130.70, 125.19, 122.33, 121.75, 114.33, 65.95, 64.79, 56.79, 56.20, 55.62, 50.22, 42.34, 39.80, 39.53, 39.10, 37.25, 36.88, 36.21, 35.79, 33.97, 31.96, 31.92, 29.70, 29.36, 28.44, 28.23, 28.01, 24.29, 23.84, 22.80, 22.68, 22.55, 21.09, 19.38, 18.72, 14.10, 11.86.

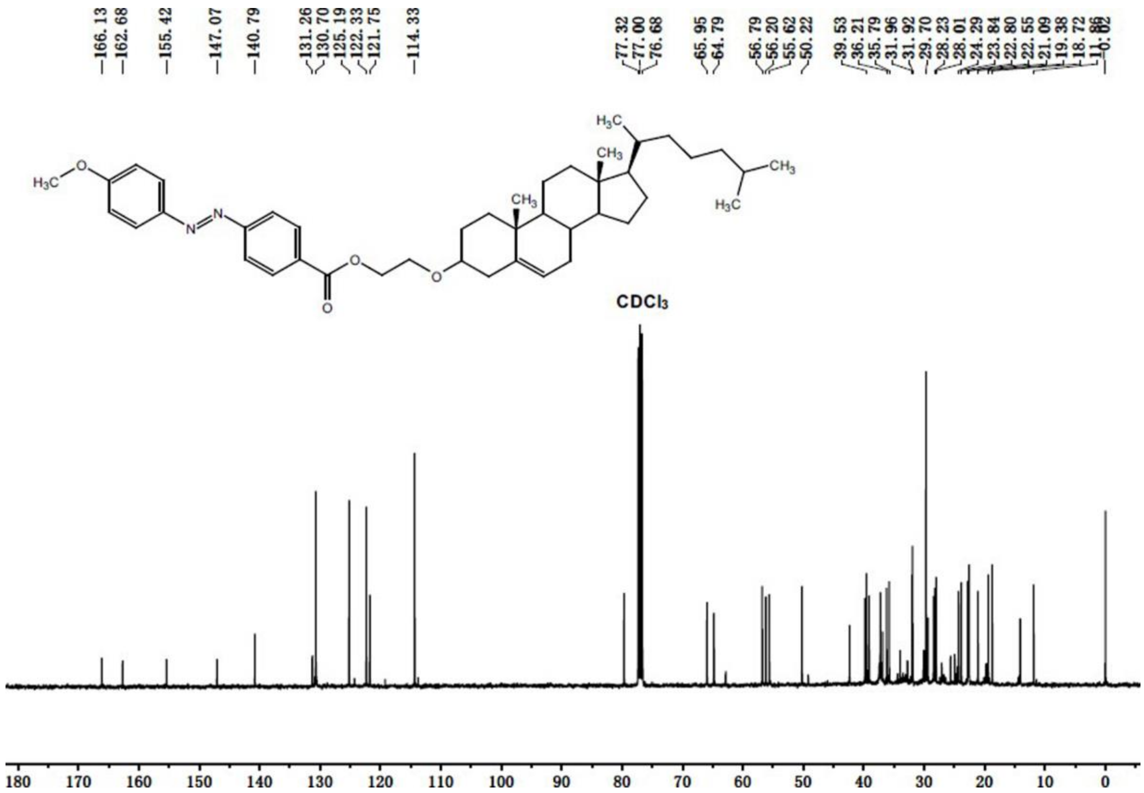

^{1}H NMR of M$_3$: ^{1}H NMR (400 MHz, $CDCl_3$) δ 8.17 (d, *J* = 8.3 Hz, 2H), 7.96 (d, *J* = 8.7 Hz, 2H), 7.90 (d, *J* = 8.3 Hz, 2H), 7.03 (d, *J* = 8.8 Hz, 2H), 5.34 (d, *J* = 4.4 Hz, 1H), 4.46 (t, *J* = 6.2 Hz, 2H), 3.91 (s, 3H), 3.65 (t, *J* = 6.1 Hz, 2H), 3.16 (m, *J* = 15.3, 5.5 Hz, 1H), 2.37– 1.03 (m, 30H), 1.00 (s, 3H), 0.91 (d, *J* = 6.4 Hz, 3H), 0.86 (d, *J* = 6.5 Hz, 6H), 0.67 (s, 3H).

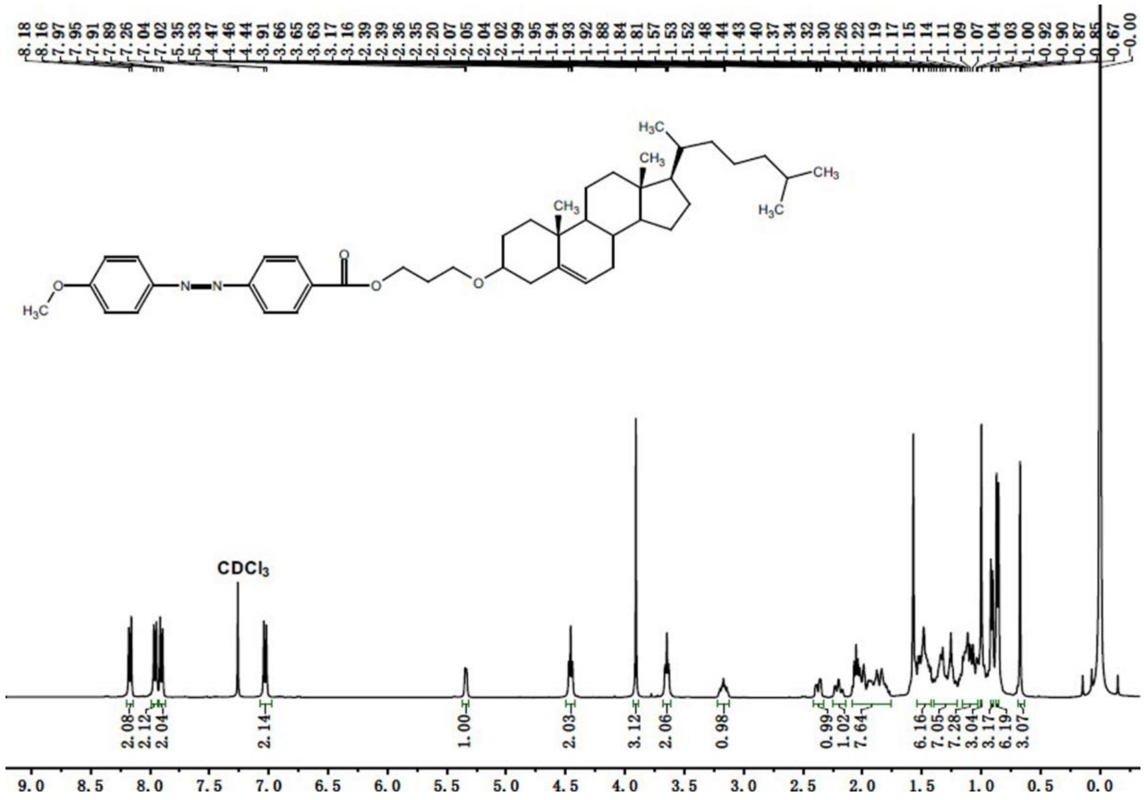

^{13}C NMR of M$_3$: ^{13}C NMR (101 MHz, $CDCl_3$) δ 166.08, 162.66, 155.34, 147.04, 140.92, 131.47, 130.53, 125.17, 122.33, 121.56, 114.31, 79.22, 64.39, 62.58, 56.78, 56.18, 55.59, 50.22, 42.32, 39.80, 39.52, 39.13, 37.24, 36.89, 36.20, 35.77, 31.94, 31.90, 29.60, 28.42, 28.22, 28.00, 24.28, 23.83, 22.79, 22.55, 21.07, 19.36, 18.71, 11.84.

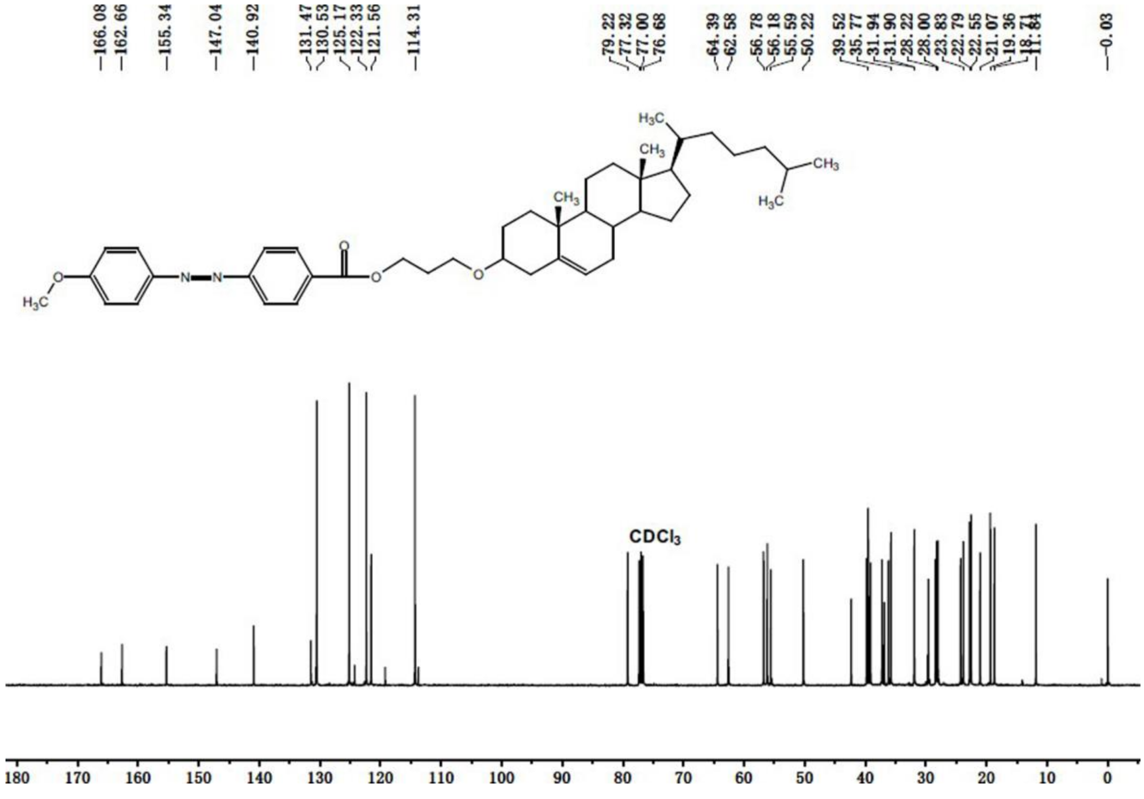

^{1}H NMR of M$_5$: ^{1}H NMR (400 MHz, $CDCl_3$) δ 8.17 (d, *J* = 8.3 Hz, 2H), 7.96 (d, *J* = 8.8 Hz, 2H), 7.90 (d, *J* = 8.3 Hz, 2H), 7.03 (d, *J* = 8.8 Hz, 2H), 5.33 (d, *J* = 4.5 Hz, 1H), 4.36 (t, *J* = 6.5 Hz, 2H), 3.91 (s, 3H), 3.50 (t, *J* = 6.2 Hz, 2H), 3.13 (m, *J* = 15.4, 5.5 Hz, 1H), 2.36– 1.00 (m, 34H), 0.99 (s, 3H), 0.91 (d, *J* = 6.4 Hz, 3H), 0.86 (d, *J* = 6.5 Hz, 6H), 0.67 (s, 3H).

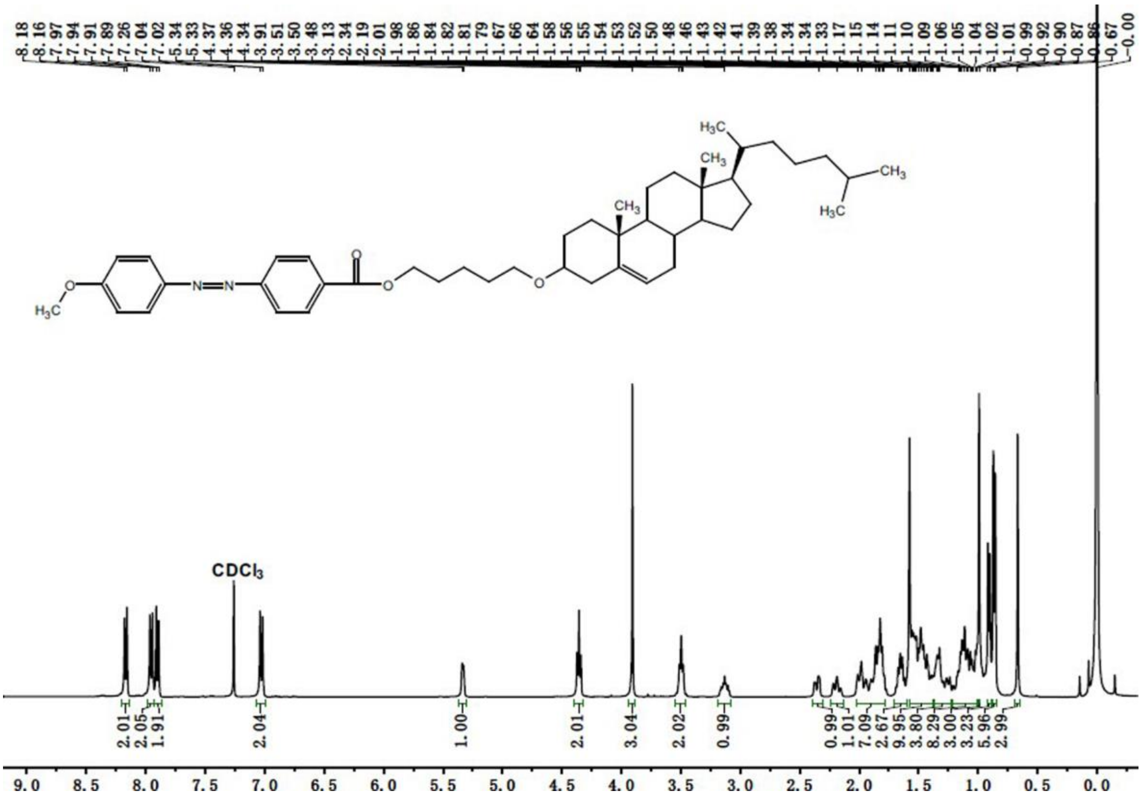

^{13}C NMR of M_5: ^{13}C NMR (101 MHz, $CDCl_3$) δ 166.12, 162.64, 155.31, 147.04, 141.05, 131.54, 130.52, 125.16, 122.32, 121.43, 114.30, 79.02, 67.69, 65.20, 56.77, 56.18, 55.58, 50.22, 42.31, 39.79, 39.51, 39.22, 37.28, 36.89, 36.19, 35.77, 31.94, 31.90, 29.84, 28.59, 28.50, 28.21, 27.99, 24.27, 23.84, 22.86, 22.79, 22.54, 21.06, 19.35, 18.71, 11.83.

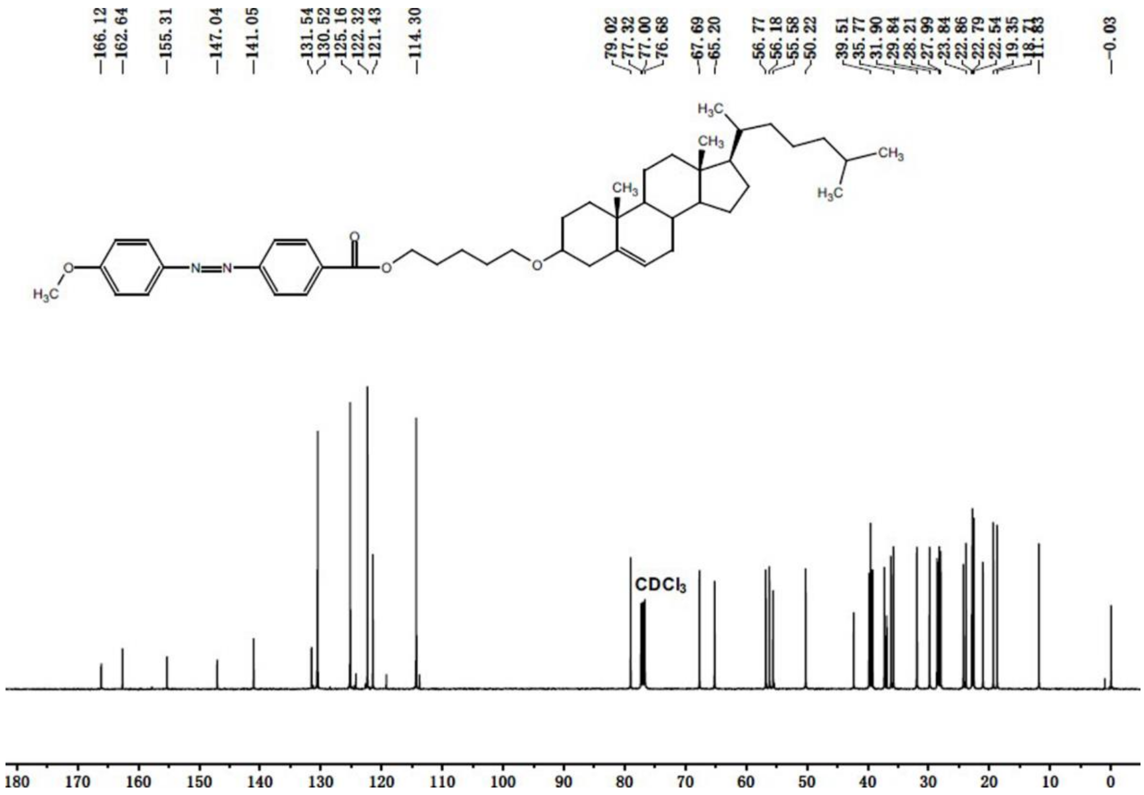

^{1}H NMR of M_6: ^{1}H NMR (400 MHz, $CDCl_3$) δ 8.17 (d, *J* = 8.6 Hz, 2H), 7.96 (d, 2H), 7.90 (d, *J* = 8.6 Hz, 2H), 7.03 (d, *J* = 9.0 Hz, 2H), 5.33 (d, *J* = 5.2 Hz, 1H), 4.35 (t, *J* = 6.5 Hz, 2H), 3.91 (s, 3H), 3.47 (td, *J* = 6.5, 1.9 Hz, 2H), 3.11 (m, *J* = 15.5, 5.6 Hz, 1H), 2.35 – 1.02 (m, 36H), 0.99 (s, 3H), 0.91 (d, *J* = 6.5 Hz, 3H), 0.87 (dd, *J* = 6.6, 1.7 Hz, 6H), 0.66 (s, 3H).

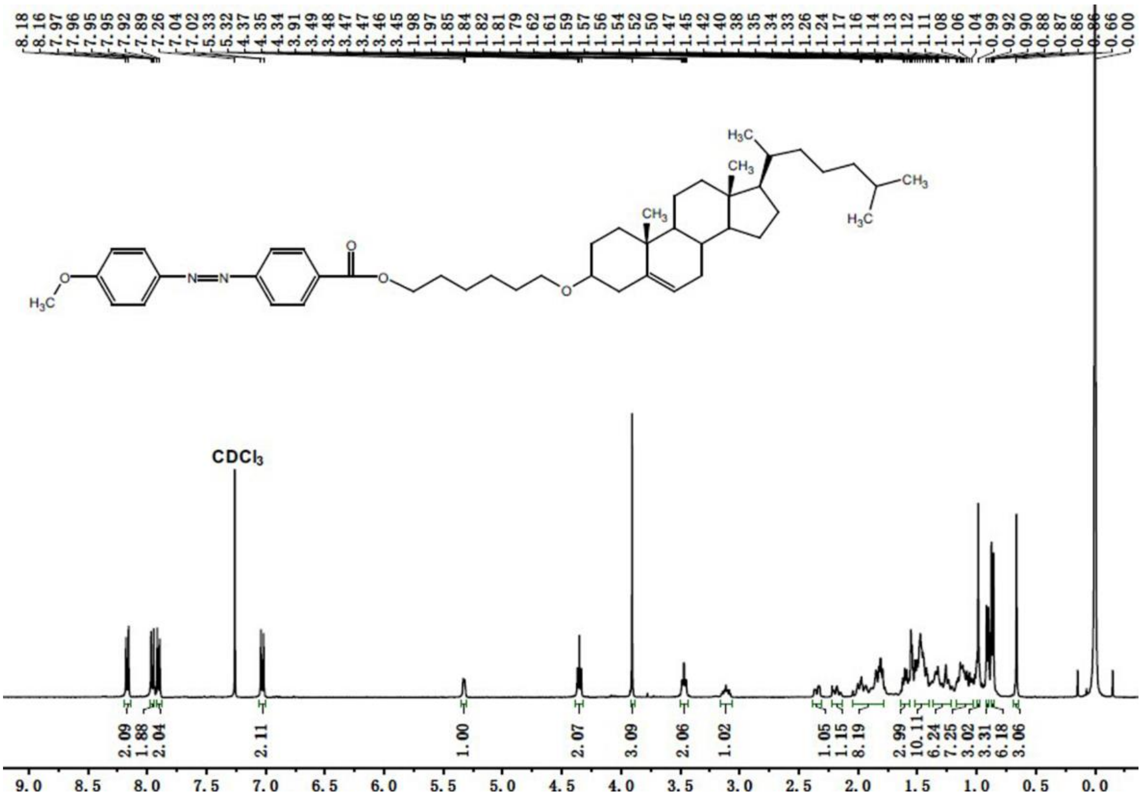

^{13}C NMR of M_6: ^{13}C NMR (101 MHz, $CDCl_3$) δ 166.13, 162.62, 155.27, 147.01, 141.05, 131.53, 130.49, 125.16, 122.33, 121.39, 114.28, 79.00, 67.80, 65.20, 56.74, 56.15, 55.57, 50.18, 42.28, 39.76, 39.50, 39.20, 37.26, 36.86, 36.18, 35.76, 31.91, 31.87, 30.03, 28.67, 28.47, 28.20, 27.98, 25.90, 24.25, 23.83, 22.79, 22.54, 21.04, 19.34, 18.69, 11.82

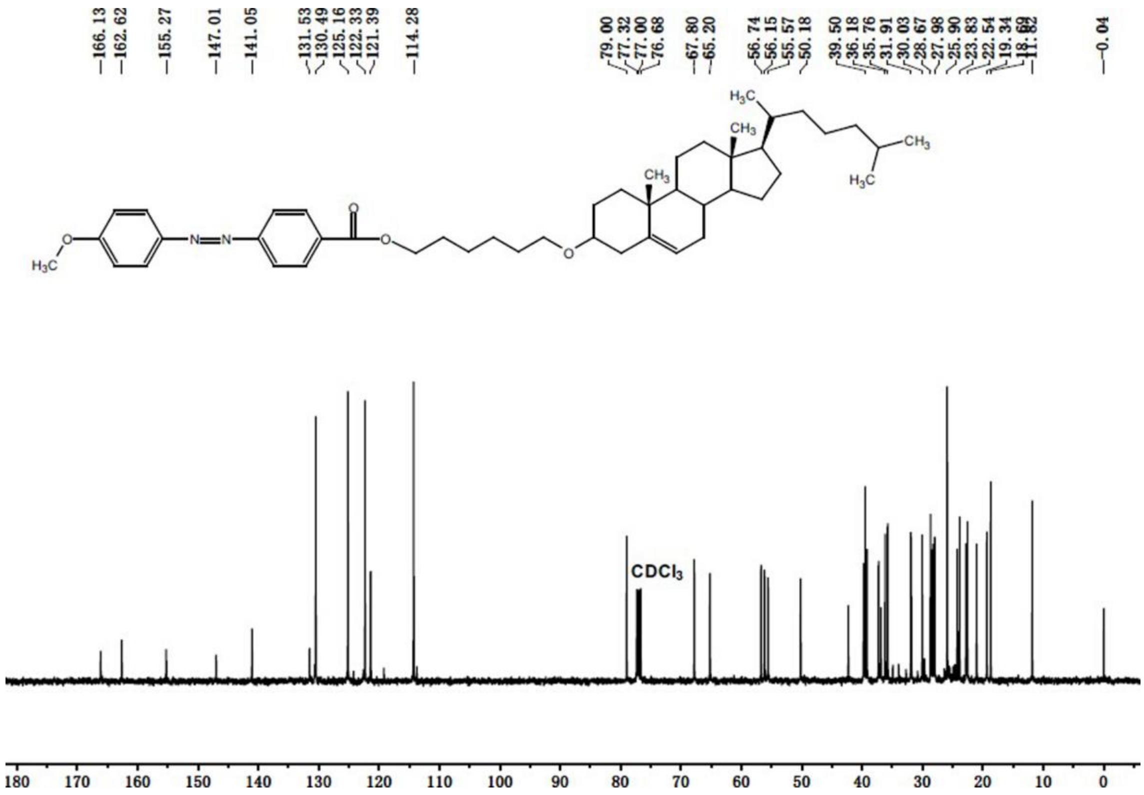

^{1}H NMR of M_{10}: ^{1}H NMR (400 MHz, $CDCl_3$) δ 8.17 (d, *J* = 8.4 Hz, 2H), 7.96 (d, *J* = 8.9 Hz, 2H), 7.90 (d, *J* = 8.4 Hz, 2H), 7.03 (d, *J* = 8.9 Hz, 2H), 5.34 (d, *J* = 4.5 Hz, 1H), 4.34 (t, *J* = 6.6 Hz, 2H), 3.91 (s, 3H), 3.44 (t, *J* = 6.6 Hz, 2H), 3.17 – 3.07 (m, 1H), 2.35 – 1.02 (m, 44H), 0.99 (s, 3H), 0.91 (d, *J* = 6.4 Hz, 3H), 0.88 – 0.85 (m, 6H), 0.67 (s, 3H).

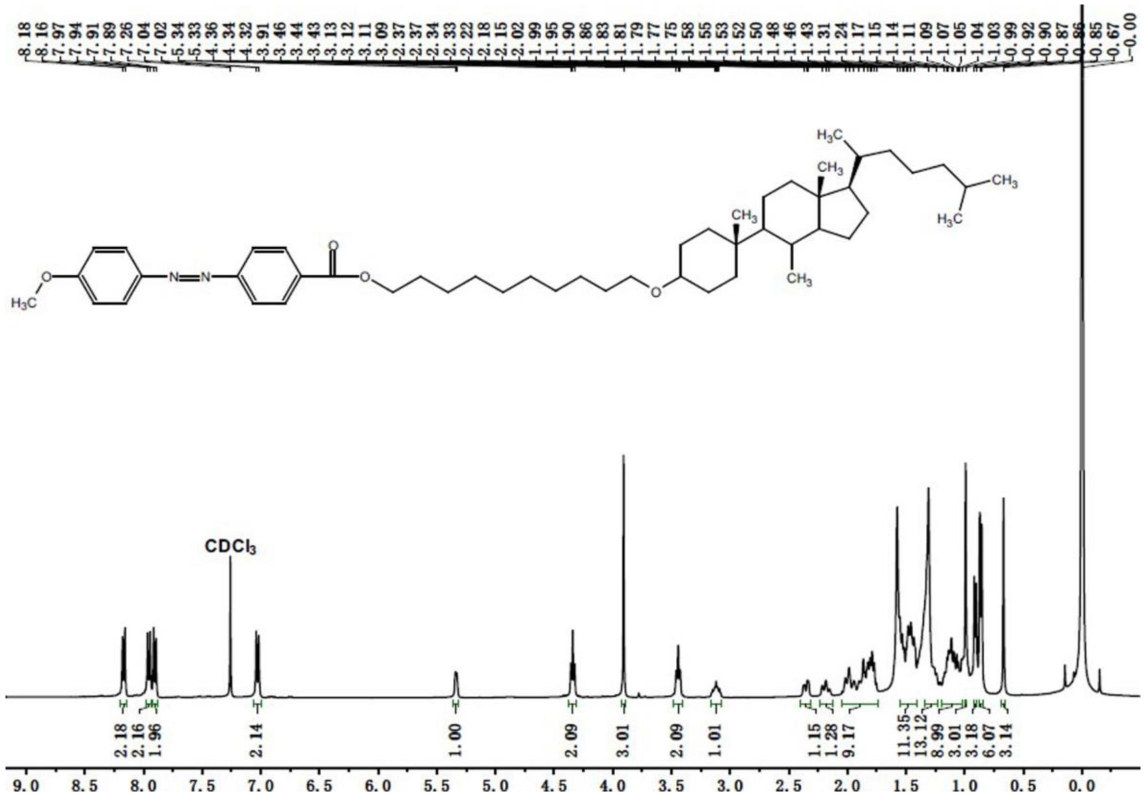

^{13}C NMR of M_{10}: ^{13}C NMR (101 MHz, $CDCl_3$) δ 166.16, 162.62, 155.28, 147.02, 141.14, 131.57, 130.49, 125.16, 122.31, 121.34, 114.28, 78.93, 68.09, 65.33, 56.76, 56.16, 55.57, 50.20, 42.30, 39.78, 39.50, 39.21, 37.29, 36.88, 36.18, 35.76, 31.93, 31.88, 30.20, 29.47, 29.42, 29.23, 28.70, 28.48, 28.21, 27.98, 26.18, 26.01, 24.26, 23.82, 22.79, 22.54, 21.05, 19.35, 18.70, 11.83.

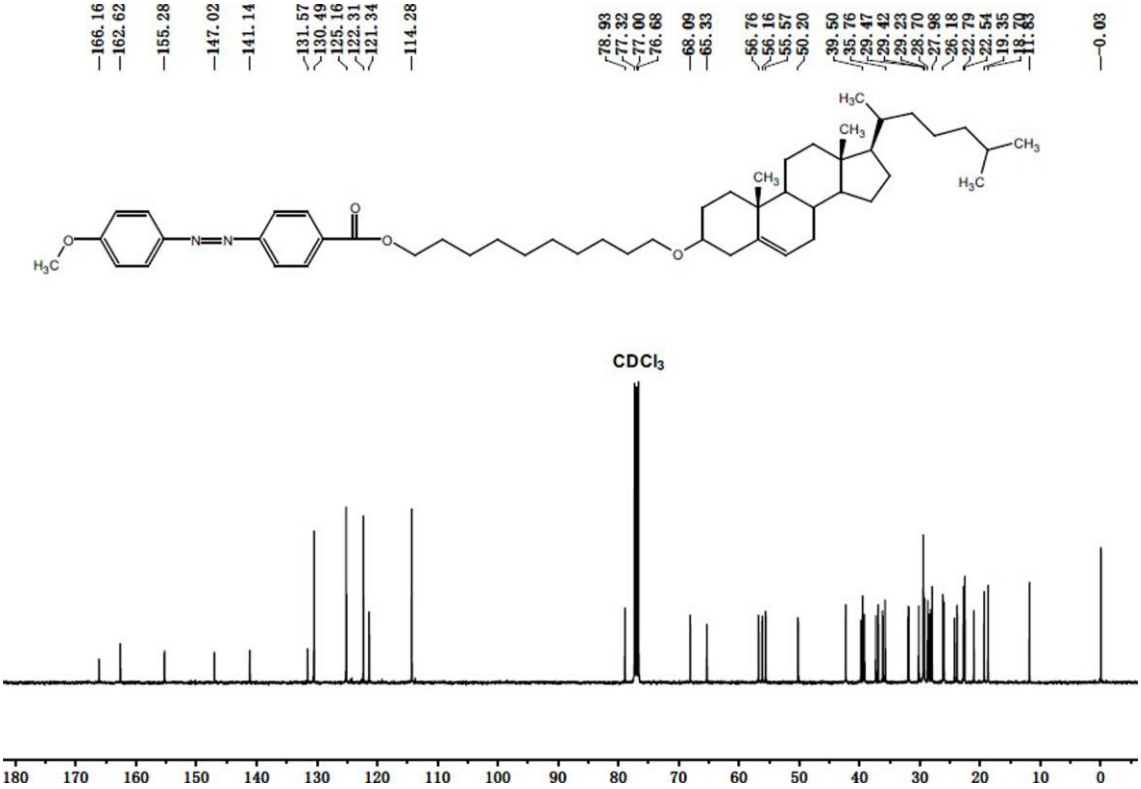

^{1}H NMR of M_{12}: ^{1}H NMR (400 MHz, $CDCl_3$) δ 8.17 (d, J = 8.4 Hz, 2H), 7.95 (d, J = 8.8 Hz, 2H), 7.90 (d, J = 8.4 Hz, 2H), 7.03 (d, J = 8.8 Hz, 2H), 5.34 (d, J = 4.6 Hz, 1H), 4.34 (t, J = 6.6 Hz, 2H), 3.90 (s, 3H), 3.44 (t, J = 6.7 Hz, 2H), 3.11 (m, J = 15.3, 5.5 Hz, 1H), 2.35– 1.01 (m, 48H), 0.99 (s, 3H), 0.91 (d, J = 6.4 Hz, 3H), 0.86 (d, J = 6.4 Hz, 6H), 0.67 (s, 3H).

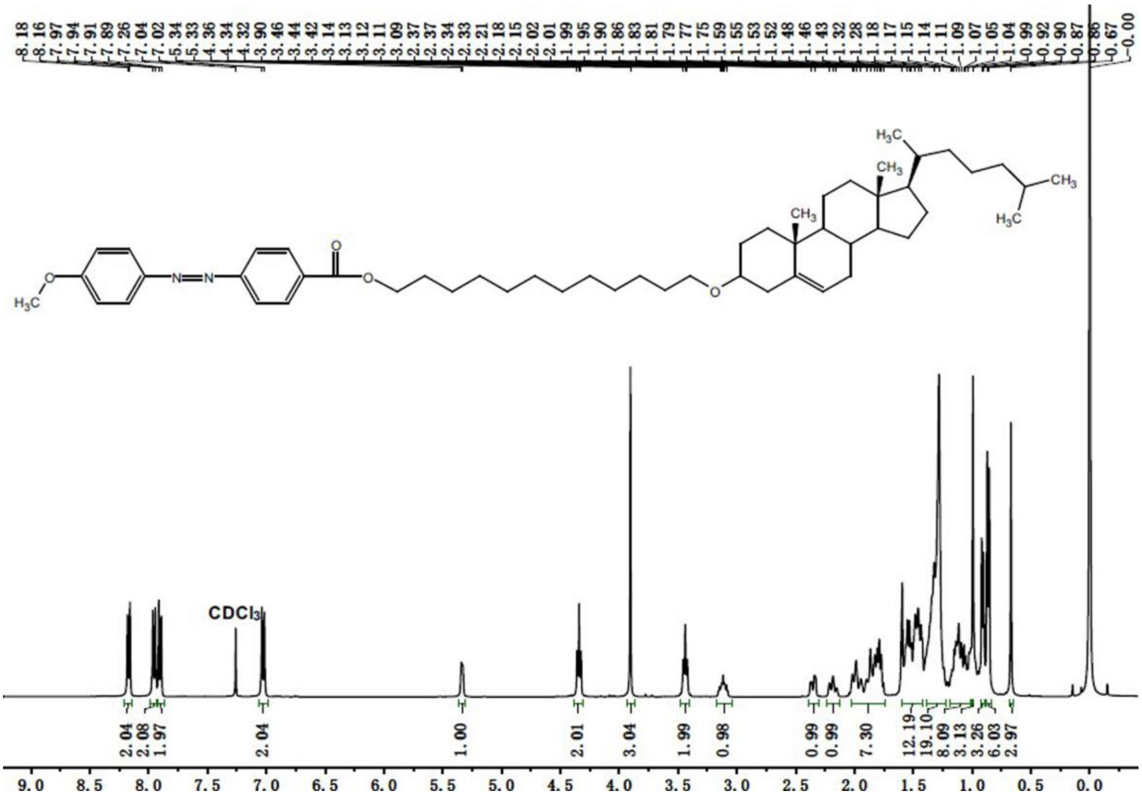

^{13}C NMR of M$_{12}$: ^{13}C NMR (101 MHz, $CDCl_3$) δ 166.14, 162.61, 155.27, 147.01, 141.14, 131.56, 130.48, 125.15, 122.30, 121.33, 114.27, 78.91, 68.11, 65.33, 56.76, 56.16, 55.56, 50.20, 42.29, 39.77, 39.50, 39.21, 37.29, 36.87, 36.17, 35.76, 31.92, 31.88, 30.21, 29.55, 29.53, 29.48, 29.26, 28.70, 28.48, 28.20, 27.98, 26.20, 26.02, 24.26, 23.83, 22.79, 22.54, 21.04, 19.35, 18.70, 11.82.

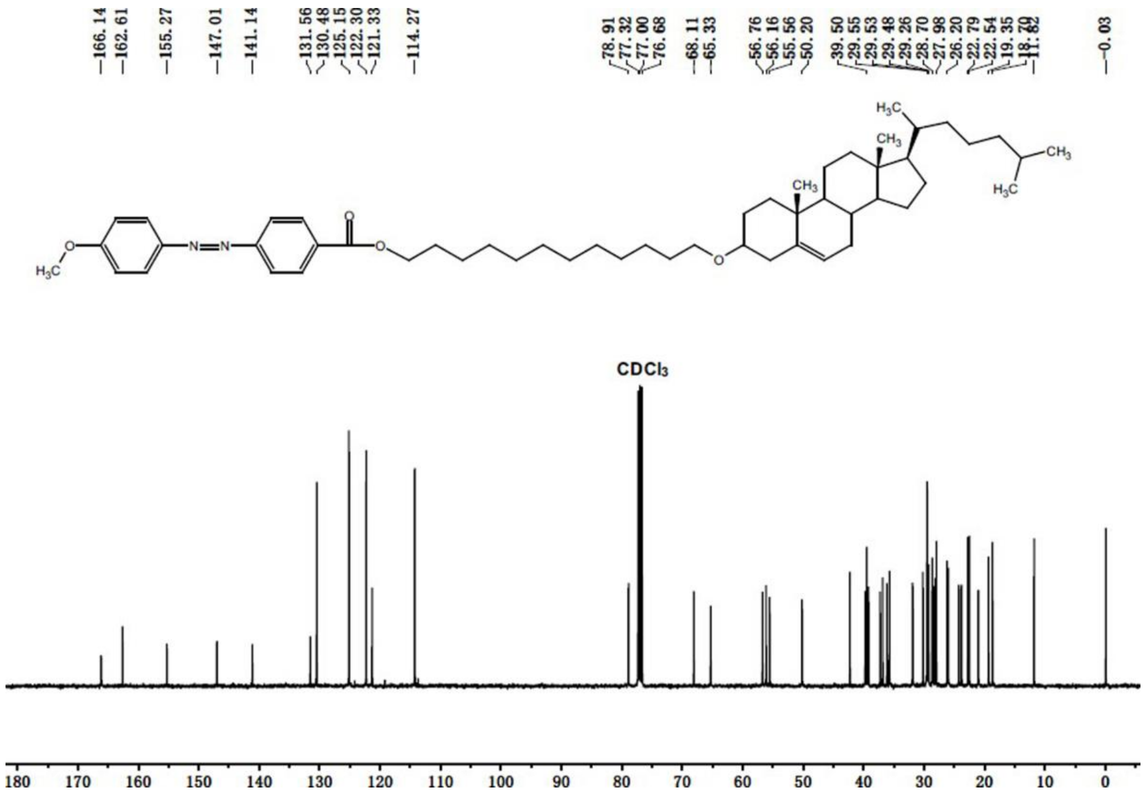

Supplement: File 1 — Spectroscopical and analytical data. [file Beilstein_J_Org_Chem-11-1089-s001.pdf]
